# Local redox conditions in cells imaged via non-fluorescent transient states of NAD(P)H

**DOI:** 10.1038/s41598-019-51526-w

**Published:** 2019-10-21

**Authors:** Johan Tornmalm, Elin Sandberg, Mihailo Rabasovic, Jerker Widengren

**Affiliations:** 1 Experimental Biomolecular Physics, Department of Applied Physics, Royal Institute of Technology (KTH), Albanova University Center, 106 91 Stockholm, Sweden; 2 Institute of Physics, University of Belgrade, Pregrevica 118, Belgrade, 11080 Serbia

**Keywords:** Multiphoton microscopy, Fluorescence spectroscopy, Biological fluorescence, Cellular imaging

## Abstract

The autofluorescent coenzyme nicotinamide adenine dinucleotide (NADH) and its phosphorylated form (NADPH) are major determinants of cellular redox balance. Both their fluorescence intensities and lifetimes are extensively used as label-free readouts in cellular metabolic imaging studies. Here, we introduce fluorescence blinking of NAD(P)H, as an additional, orthogonal readout in such studies. Blinking of fluorophores and their underlying dark state transitions are specifically sensitive to redox conditions and oxygenation, parameters of particular relevance in cellular metabolic studies. We show that such dark state transitions in NAD(P)H can be quantified via the average fluorescence intensity recorded upon modulated one-photon excitation, so-called transient state (TRAST) monitoring. Thereby, transitions in NAD(P)H, previously only accessible from elaborate spectroscopic cuvette measurements, can be imaged at subcellular resolution in live cells. We then demonstrate that these transitions can be imaged with a standard laser-scanning confocal microscope and two-photon excitation, in parallel with regular fluorescence lifetime imaging (FLIM). TRAST imaging of NAD(P)H was found to provide additional, orthogonal information to FLIM and allows altered oxidative environments in cells treated with a mitochondrial un-coupler or cyanide to be clearly distinguished. We propose TRAST imaging as a straightforward and widely applicable modality, extending the range of information obtainable from cellular metabolic imaging of NAD(P)H fluorescence.

## Introduction

Cellular redox balance is central to the regulation of energy production and intermediary metabolism, as well as for cell survival, growth and proliferation. Alterations in this balance have been coupled to a broad range of pathological conditions, including neurodegenerative, infectious and inflammatory diseases, and cancer^1–3^. Two major determinants of cellular redox balance are the nicotinamide adenine dinucleotide (NAD^+^/NADH) and nicotinamide adenine dinucleotide phosphate (NADP^+^/NADPH) redox couples. The primary role of NAD^+^ is to act as an electron acceptor in catabolic pathways, while NADPH acts as a central electron donor in anabolic pathways^4^. Both NADH and NADPH, hereinafter referred to as NAD(P)H, are fluorescent, while their oxidized forms, NAD(P)^+^, are not.

NAD(P)H autofluorescence, typically studied by laser scanning microscopy (LSM) and two-photon excitation (TPE)^5^, offers a versatile readout for label-free metabolic imaging of cells and tissues^6–8^. Several relations between metabolic cellular phenotypes and the recorded fluorescence properties of NAD(P)H can be exploited. By optical redox ratioing (ORR), comparing cellular fluorescence intensity of NAD(P)H with that of oxidized flavin adenine dinucleotide (FAD^+^), it is possible to estimate the balance between oxidative phosphorylation and glycolysis for adenosine triphosphate (ATP) production in cells^9,10^. However, inhomogeneous intra-cellular concentrations of NAD(P)H and FAD^+^, together with local variations in fluorescence quantum yield due to enzyme binding^11^, makes plain intensity quantification of cellular metabolic states difficult. Fluorescence lifetime microscopy (FLIM) can account for these effects and determine the ratio of free to protein-bound NAD(P)H by the fraction of short (*τ*_*free*_) and long (*τ*_*bound*_) fluorescence lifetimes in the sample. This free-vs-bound ratio has been found to be a sensitive indicator of cellular energy metabolism^12,13^, capable to identify metabolic phenotypes representing early pathological conditions in cells^7,14–17^, mitochondria and other subcellular compartments^18^. Moreover, local variations in *τ*_*bound*_, attributed to binding of NAD(P)H to different enzymes and conformational heterogeneities of those enzymes, have been reported to reflect different cellular metabolic states^19^. Still, despite numerous successful examples of the use of NAD(P)H fluorescence and TPE FLIM to non-invasively monitor cellular metabolism, identify metabolic cellular phenotypes, and the many potential clinical applications of such assessments ^8,20^, the biochemical basis for variations in ORR, free-vs-bound NAD(P)H, and *τ*_*bound*_ is not yet fully understood^11^. The assessments are influenced by artifacts and give only a relatively limited view of the metabolic states of the cells. NADH and NADPH show almost identical fluorescence properties in aqueous solution^21,22^, and have been found difficult to separately detect inside cells^23^. The two co-enzymes take part in several, distinctly different metabolic pathways^4^, and changes in the redox balance within cells may thus lead to different changes in the reduced, fluorescent fractions of each of these compounds, and in their protein-bound fractions. This complicates the interpretation of changes in the NAD(P)H fluorescence readouts in cells^11^, and changes in NADH and NADPH fluorescence may even cancel each other out.

One way to overcome current limitations in label-free cellular metabolic imaging is to use additional, orthogonal information in the NAD(P)H fluorescence. Such information can be obtained from the fluorescence blinking of NAD(P)H, upon transitions to and from photo-induced dark states. Studies by fluorescence correlation spectroscopy (FCS) and other single-molecule techniques have shown that blinking properties of organic fluorophores, generated by transitions to and from dark triplet and photo-oxidized states, are highly sensitive to the immediate environment around the fluorophores^24,25^. Notably, these transitions are specifically sensitive to redox conditions and oxygenation, parameters of particular relevance in cellular metabolic studies. The photophysics underlying such blinking properties in NAD(P)H have been extensively studied, in particular by transient absorption and electron spin resonance (ESR) spectroscopy^26–29^. However, although these methods have proven useful for the photophysical characterization of NAD(P)H itself, they do not lend themselves to studies in live cells. FCS is compatible with live cell studies, but requires single-molecule detection conditions. The fluorescence brightness of individual NAD(P)H molecules is too weak for FCS studies, in any sample medium.

Here, we show that the major photophysical transitions of NAD(P)H can be determined by monitoring its fluorescence intensity in response to systematic modulation of a laser excitation source, by so-called transient state (TRAST) spectroscopy/imaging^30,31^. We first characterized dark-state transitions of NADH in aqueous solution, and demonstrate that, by this simple and widely applicable method, we can obtain the same data as from transient state absorption and ESR spectroscopy. We then show that these transitions can be imaged on a local, subcellular scale in live cells, providing information on redox environments and metabolic states not clearly reflected in traditional fluorescence parameters. The proposed procedure circumvents all major limitations of FCS and related single-molecule techniques to assess NAD(P)H dark state transitions in live cells and can be applied in parallel with standard TPE FLIM assessments.

## Results

From TRAST measurements on NADH in aqueous solution using one-photon excitation (OPE) in the UV, a photophysical model for NADH was established, tested under different experimental conditions, and compared to literature^26–29^. Based on this model, we investigated the photophysical properties of NADH in aqueous solution upon TPE, with an IR laser systematically scanned with different speeds across the sample. Thereby, we could determine a slightly modified photophysical model for NADH under TPE conditions. Using a similar laser-scanning approach, we finally demonstrated TRAST imaging of NAD(P)H in live cells, and that orthogonal information about metabolic states of cells can be obtained, assessed in parallel with the full toolbox of TPE FLIM.

### Solution measurements with one-photon excitation

TRAST measurements were performed on NADH in aqueous solution, using a confocal setup with a stationary 355 nm laser beam for OPE. The excitation laser was intensity-modulated, so that samples were subject to square-wave excitation pulse trains of varying duration, *w* (see Methods and Materials). We analysed the photophysics of NADH under different environmental and excitation conditions by so-called TRAST curves^30,32,33^, showing the normalized time-averaged fluorescence of NADH, 〈*F*_*exc*_(*w*)〉_*norm*_, as a function of pulse duration, *w*. By varying the measurement conditions to specifically affect one or several of the major photo-induced states of NADH, and guided by previous photophysical studies of NADH by transient absorption and ESR spectroscopy^26–29^, we could define a photophysical model for NADH relevant for our measurement conditions. This model was verified by global fitting of all the TRAST curves, whereby also the rate parameters for the photophysical transitions of NADH were determined. (Hereinafter, stated confidence intervals for determined rate parameter values and slopes are 95% confidence intervals).

#### Effects of excitation intensity and oxygen concentration

**Figure 1:**
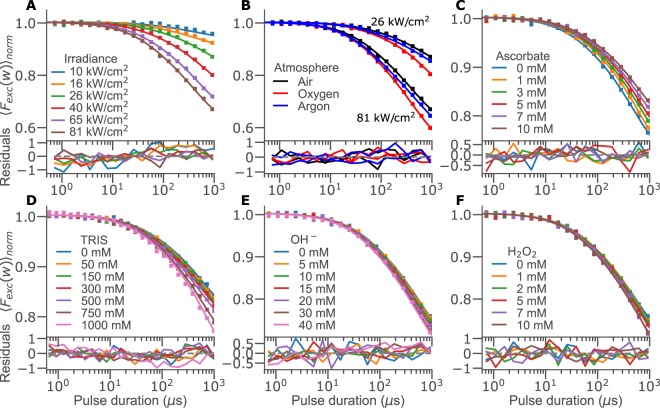
Experimental TRAST curves recorded from 1 *μM* NADH (50 mM TRIS, pH 7.4, if not stated otherwise) under OPE. (**A**) Irradiance (*I*_*exc*_) dependence (10 to 81 kW/cm^2^). (**B**) Influence of oxygen on the TRAST curves (only two irradiances shown for clarity). Titrations of (**C**) sodium ascorbate, (**D**) TRIS buffer at pH 7.4, (**E**) sodium hydroxide in aqueous solution without TRIS buffer and (**F**) H_2_O_2_ (measured at 50 kW/cm^2^). All curves (A-F, solid lines) were fitted simultaneously (see Global parameter fitting to TRAST curves generated under OPE) and are shown with absolute residuals, multiplied by a factor of 100 for clarity.

Figure 1A shows TRAST curves recorded from an air-saturated solution of NADH, under different excitation intensities. The curves are approximately flat in the time range of 500 ns to 10 μs, indicating little or no triplet state population present within the typical time scale for triplet state relaxation, as observed for organic fluorophores^24^ and autofluorescent compounds^32,33^. For pulse durations, *w*, from 10 μs up to 1 ms, a prominent dark state build-up can be noted. The time-scale and multi-exponential behaviour of this decay, resulting in an almost linearly declining TRAST curve, is compatible with stepwise photo-oxidation of NADH to its non-fluorescent form NAD^+^.

To support these interpretations, we repeated the measurements in atmospheres of pure oxygen and pure argon (Fig. 1B). Oxygen-saturated measurements revealed an increased decay in the TRAST curves on a time scale of around 100 μs. Given the minimal triplet state population already under air-saturated conditions (Fig. 1A), the triplet state can be neglected entirely in a pure atmosphere of oxygen, a potent triplet state quencher. The increased decay is instead attributed to an increased build-up of photo-oxidized NADH, promoted by the presence of more oxygen. Complete de-oxygenation by argon also resulted in a minor increase of the TRAST curve decay, but on a faster time-scale. This effect is compatible with the presence of a minor triplet state population, barely observable under air-saturated conditions.

#### Photophysical model for NADH

**Figure 2:**
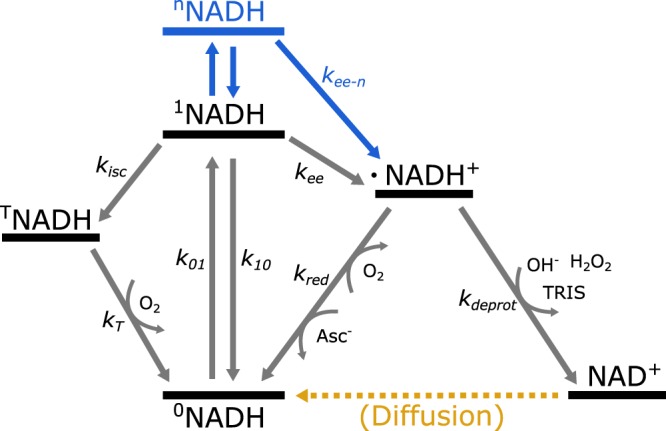
Photophysical model for NADH, adapted for TRAST analysis. Excitation takes place from the singlet ground state (^0^NADH) to the first excited singlet state (^1^NADH), or to a higher excited singlet state (^n^NADH). Excitation can result in dark state formation, via intersystem crossing to a triplet state (^T^NADH), or via electron-ejection into a radical cation (**·**NADH^+^). **·**NADH^+^ rapidly deprotonates into a neutral radical, **·**NAD^40^, followed by a second one-electron oxidation to form the stable oxidized form NAD^+^. As a competing route, **·**NADH^+^ can also return to ^0^NADH, by recombining with a free electron, or taking up an electron from an electron donor. The short-lived **·**NAD radical was not explicitly included in the model, since although it can also dimerize, leading to a manifold of other reactions^57^, the one dominating process under our experimental conditions is the further oxidation to NAD^+^. Rate parameters: ground state excitation rate (*k*_01_), combined fluorescence and non-radiative decay rate (*k*_10_), intersystem crossing rate (*k*_*isc*_), triplet relaxation rate (*k*_*T*_), electron ejection rate from ^1^NADH (*k*_*ee*_), electron ejection rate from ^n^NADH (*k*_*ee*−*n*_), radical deprotonation rate (*k*_*deprot*_), radical reduction rate (*k*_*red*_). The blue part of the model refers to transitions which under our experimental conditions only took place with TPE, as discussed in the main text. The yellow part of the model represents diffusion mediated recovery of ^0^NADH in the excitation volume of the experiment (see SI section 4).

The observations in the TRAST curves, as shown in Fig. 1A,B, are well in line with previous photophysical studies of NADH. With optical detection of magnetic resonance (ODMR) and photoluminescence studies, no^34^, or very minor^35^ evidence of triplet state formation in NADH could be observed upon excitation at 313 nm or longer wavelengths. In contrast, transient state absorption^26,27,29^ and ESR^28,36^ studies show that radical cations of NADH are readily formed upon light excitation. Based on the effects of excitation intensity and oxygen concentration observed with TRAST (Fig. 1A,B), and taking these previous studies of NADH photophysics^26–29,34–36^ into account, we constructed a photophysical model for NADH (Fig. 2). The purpose of the model is to include all dark state transitions captured in our TRAST measurements (but to leave out details of dark states, such as tautomerizations, two electron ejection, multiple protonation and dimerization states, which cannot be distinguished from each other by our TRAST measurements).

In the model of Fig. 2, the first step of NADH photo-oxidation is the ejection of a single electron, forming the radical cation **·**NADH^+^. Some controversy remains in literature whether this electron ejection has a one-photon^27^ or a step-wise two-photon^26,29^ excitation dependence, and under what conditions the two different electron ejection models would apply. To investigate which model is suitable for our TRAST measurements, we studied how the decay amplitudes, *A*_*TRAST*_, of the TRAST curves were related to the applied excitation irradiance, *I*_*exc*_. *A*_*TRAST*_ represents the total dark state build-up in NADH for long *w*. In absence of excitation saturation (${k}_{01}\ll {k}_{10}$) and neglecting any triplet state formation, the effective photo-oxidation rate from ^0^NADH to NAD^+^ is ${k}_{ox}^{\text{'}}\propto {A}_{TRAST}/(1-{A}_{TRAST})$. A log-log plot of *A*_*TRAST*_/(1 − *A*_*TRAST*_) versus *I*_*exc*_ produces a straight line with a slope of 1.06 ± 0.1 (data not shown), suggesting a predominantly linear *I*_*exc*_-dependence under our experimental conditions. A similar analysis of initial fluorescence intensity (i.e. the fluorescence intensity recorded for short *w*, with negligible dark state build-up) versus *I*_*exc*_ gives a slope of 0.97 ±0.05 (data not shown), confirming that ${k}_{01}\ll {k}_{10}$ over the range of *I*_*exc*_ used.

Finally, we performed TRAST measurements (43 kW/cm^2^) with additions of up to 10 mM potassium iodide (KI), known to affect both intersystem crossing and triplet state quenching in many fluorophores^37^. However, no changes in *A*_*TRAST*_ could be found under our experimental conditions. This suggests that triplet state formation in NADH is minor and that electron ejection from the triplet state can be neglected as a pathway for **·**NADH^+^ formation.

#### Redox, pH and buffer dependence

To confirm the model of Fig. 2, and to investigate the sensitivity of TRAST measurements to biological factors such as pH, redox balance and buffer strength, we performed TRAST experiments with sodium ascorbate (anti-oxidant, 0–10 mM), hydrogen peroxide (oxidant, 0–10 mM), sodium hydroxide (pH, 0–40 mM) or TRIS buffer (0–1 M) added into the NADH solution.

After formation of the radical cation **·**NADH^+^, fast deprotonation (by a concerted electron-H atom transfer from **·**NADH^+^) is known to follow, at first-order rates in the range of 10^6^ s^−1^^29^. **·**NADH^+^ is strongly acidic and the deprotonation from **·**NADH^+^ to NAD^+^ is essentially irreversible under biologically relevant conditions^38,39^. However, the fact that reported values for the NADH electron ejection yields are higher than the corresponding photo-oxidation yields^27^, suggests that **·**NADH^+^ can also recombine with a solvated electron, thereby returning to ^0^NADH. We investigated this recombination by adding ascorbate (Asc^−^) as an electron donor, which was found to decrease the decay amplitudes in the TRAST curves (Fig. 1C), consistent with promoted recombination of **·**NADH^+^ back to ^0^NADH. However, the decrease was lower than for other auto-fluorescent compounds^32^ or fluorophores^25^. This can be explained by the comparably short lifetime of **·**NADH^+^, and that reduction back to ^0^NADH competes with rapid deprotonation and further oxidation to NAD^+^.

In contrast to Asc^−^, addition of hydrogen peroxide (H_2_O_2_), hydroxide ions (OH^−^) or TRIS buffer slightly increased the decay amplitude of the recorded TRAST curves (Fig. 1D–F). This indicates an increased formation of NAD^+^, with the compounds acting as acceptors of either protons (TRIS), or electron-H atom units^38,39^. The relatively weak effects seen in the TRAST curves upon addition of these compounds supports the view that they have a minor relative effect on the fast deprotonation rate of **·**NADH^+^^29^.

We also investigated if NADPH shows different photo-oxidation dark state transitions compared to NADH. However, no significant differences could be observed under the experimental conditions applied for the NADH studies described above.

#### Global parameter fitting to TRAST curves generated under OPE

Based on the rate equations following from the photophysical model of NADH (Fig. 2), and by use of eq. (S3) with simplifications according to (S10) and (S12), rate parameters were numerically fitted (see Methods and Materials) to all the recorded TRAST curves described above. In the fit, the excitation rate (${k}_{01}=\sigma {\Phi }_{exc}$) was set based on a reported excitation cross section, σ, of 2.0 · 10^−17^ cm^2^ (at 355 nm)^21^ and a local photon flux calculated as ${\Phi }_{exc}={I}_{exc}\lambda /(hc)$, where *hc*/*λ* is the photon energy. All other rates (*k*_*isc*_, *k*_*T*_, *k*_*ee*_ *k*_*red*_, *k*_*deprot*_ and diffusion recovery parameters) were fitted globally (to same values for all 53 TRAST curves). Linear concentration dependencies were assumed for *k*_*T*_ (O_2_), *k*_*red*_ (O_2_ and Asc^−^), and *k*_*deprot*_ (H_2_O_2_, OH^−^ and TRIS buffer). A constraint was added on the combined ^1^NADH decay rates (*k*_10_ + *k*_*isc*_ + *k*_*ee*_ = 1/*τ*_*F*_) to reproduce the experimental fluorescence lifetime *τ*_*F*_ = 0.4 ns^21,27^.

Fitted curves with residuals are shown in Fig. 1A–F, and the fitted parameter values in Table 1. The intersystem crossing rate, *k*_*isc*_, was determined to 0.9 · 10^6^ s^−1^, which is comparable to *k*_*isc*_ rates of organic fluorophores with low triplet quantum yields, Ф_T_^24^. However, since *τ*_*F*_ of NADH is approximately an order of magnitude shorter than for such fluorophores, its Ф_T_ is also lower (~0.0004), which explains the very minor triplet state build-up noticed in the TRAST curves, and reported difficulties to detect ^T^NADH formation by e.g. flash photolysis^26^. The decay rate of ^T^NADH, *k*_*T*_, is largely due to diffusion-controlled quenching by molecular oxygen, and is comparable to *k*_*T*_ rates of organic fluorophores^24^ and other autofluorescent compounds^32,33^. The photo-induced electron ejection rate, *k*_*ee*_, was determined to 5.3 · 10^6^ s^−1^, orders of magnitude higher than photo-oxidation rates for organic fluorophores under similar conditions^25^, but comparable to that of tryptophan^32^. On the other hand, the resulting electron ejection quantum yield, ${\Phi }_{ee} \sim 0.002$, is much lower than those determined by flash photolysis^26,27^, ${\Phi }_{ee} \sim 0.08-0.4$. This suggests that a large fraction of the ejected electrons recombine with ∙NADH^+^, thereby forming ^0^NADH, before full dissociation from ∙NADH^+^. Flash photolysis studies show that this recombination takes place on a time scale of pico- to nanoseconds^26^. In our model, on the slower time scale reflected in the TRAST measurements, the combined electron ejection and recombination process is therefore contained in the overall decay rate, *k*_10_, from ^1^NADH to ^0^NADH. ∙NADH^+^ molecules not directly recombining with their ejected electrons can either return to ^0^NADH by taking up another electron from the solvent molecules (*k*_*red*_), or undergo de-protonation by a concerted electron-H atom transfer^38^ into NAD^+^ (*k*_*deprot*_). From the TRAST data, *k*_*deprot*_ was determined to 3.9 · 10^6^s^−1^, which agrees well with literature data^29,40^. Given its nature, it is reasonable that *k*_*deprot*_ is increased upon addition of OH^−^, TRIS and H_2_O_2_, as observed. Second order rate constants of *k*_*deprot*_ have only been reported for OH^−^^40^, and is in agreement with the value found in this study (see Table 1). For the solvent-mediated reduction rate of ∙NADH^+^, *k*_*red*_, we determined a first order rate of 2.8 · 10^6^ s^−1^, and second order rate constants, *k*_*Q,red*_, of 2.5 · 10^8^ M^−1^ s^−1^ and −1.6 · 10^9^ M^−1^ s^−1^ for Asc^−^ and O_2_, respectively. The value for *k*_*Q,red*_(Asc^−^) is about an order of magnitude higher than that determined for tryptophan^32^, but correspondingly lower than that for the organic dye rhodamine 6 G^25^, under similar experimental conditions. The negative value of *k*_*Q,red*_(O_2_) likely reflects the high electron affinity of molecular oxygen, which reduces the supply of electrons available to ∙NADH^+^, for recombination back to ^0^NADH. This is in agreement with the finding that O_2_ increases the overall photo-oxidation rate of NADH to NAD^+^^41^.

**Table 1:** Rate parameter values for the NADH model in Fig. 2, as determined by global fitting of TRAST curves recorded in aqueous solution under OPE at 355 nm (Fig. 1). Confidence intervals reflect specifically the uncertainty in the global, non-linear least square fit of the TRAST curves.

Rate	Addition	Global fit	95% conf.	Unit
k_isc_	−	0.93	±0.21	*μs* ^−1^
k_T_	− *O*_2_	0.021 1.6	±0.0044 ±0.29	*μs* ^−1^ mM^−1^ *μs*^−1^
k_ee_	−	5.3	±0.49	*μs* ^−1^
k_red_	− *O*_2_ *Asc* ^−^	2.8 −1.6 0.25	±0.51 ±0.34 ±0.027	*μs* ^−1^ mM^−1^ *μs*^−1^ mM^−1^ *μs*^−1^
k_deprot_	− *TRIS* *H*_2_ *O*_2_ *OH* ^−^	3.9 0.012 0.4 0.16	±0.38 ±0.0033 ±0.23 ±0.055	*μs* ^−1^ mM^−1^ *μs*^−1^ mM^−1^ *μs*^−1^ mM^−1^ *μs*^−1^

To exclude alternative photophysical models, we also evaluated 8 variations of the model in Fig. 2 and determined the most likely candidate based on the Akaike and Bayesian information criteria (AIC and BIC)^42–44^. Indeed, the AIC and BIC scores clearly favour the model of Fig. 2, with a one-photon excitation dependence of *k*_*ee*_, which solely originates from ^1^NADH, and not from ^T^NADH, yet with ^T^NADH included in the model (See SI section 8 for details regarding the evaluated models and the resulting relative likelihoods). The NAD^+^ state in our model in reality represents several states, all non-fluorescent and long-lived. On the time-scale of the TRAST experiments the recovery rate from NAD^+^ back to ^0^NADH can thus be solely attributed to a diffusion-mediated net influx of fresh molecules, from the solution outside of the focused laser excitation beam. To account for this influx, we used two dark states with separate recovery rates in the model, representing the different influx rates along the elongated axial and shorter radial dimension of the laser-excited detection volume (see SI section 4 for details). This is also the diffusion model favoured by the AIC and BIC analysis. The effective diffusion recovery rates were kept constant throughout the global analysis and did not affect any relative changes seen in the TRAST curves.

### Solution measurements with two-photon excitation

With the photophysical model established for OPE TRAST measurements of NADH in aqueous solution, we next investigated how this model applies to TPE by a pulsed Ti:Sapphire laser beam, circularly scanned in the sample (see Methods and Materials). TRAST curves were generated by varying the scanning speed, as previously demonstrated for OPE^31^, while recording the average fluorescence intensity for each effective excitation duration, *w*_*eff*_.

#### Excitation power dependence and ascorbate titration effects

**Figure 3:**
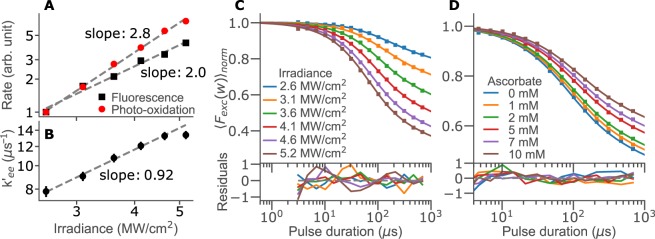
TPE TRAST measurements of 10 μM NADH in 50 mM TRIS buffer, pH 7.4. (**A**) Excitation irradiance dependence of the effective photo-oxidation (red circles) and fluorescence emission (black squares) rates, under TPE. The rates are displayed on a log-log scale, in arb. units, with slopes determined by a linear fit. (**B**) Excitation irradiance dependence of the fitted *k*_*ee*_ rates from the TRAST curves in (**C**), displayed on a log-log scale. The slope is determined by a linear fit, excluding the highest excitation intensity. (**C**) Experimental TRAST curves from NADH with different excitation irradiances applied. (**D**) Experimental TRAST curves from NADH (4.6 MW/cm^2^) with different concentrations of ascorbate added. TRAST curves in C and D were fitted as described in the main text, with absolute residuals multiplied by a factor of 100 for clarity.

TRAST curves under TPE, with time-averaged *I*_*exc*_ varying from 2.6 to 5.2 MW/cm^2^ are shown in Fig. 3C. The initial fluorescence intensity plotted against *I*_*exc*_ on a log-log scale yields a fitted slope of 2.0 ± 0.36 (Fig. 3A), in agreement with the expected TPE dependence. Plotting the effective photo-oxidation rates, $k{\text{'}}_{ox}\propto {A}_{TRAST}/(1-{A}_{TRAST})$, in the same way (Fig. 3A), yields a slope of 2.8 ± 0.2. This compares well with the power-cubed dependence reported for photo-bleaching of several fluorophores, including NADH, under similar TPE conditions^45^, but is not compatible with the linear electron ejection model used above for OPE. We therefore modified the NADH electronic state model by adding a second pathway for electron ejection, *k*_*ee−n*_, taking place via OPE of ^1^NADH to a higher electronic state, ^n^NADH. Electron ejection from ^n^NADH then takes place with a much higher yield compared to ^1^NADH, see Fig. 2 (blue addition). Similar non-linear effects on photo-bleaching and photo-oxidation have also been observed in fluorophores upon OPE at *I*_*exc*_ levels at or beyond saturation^46,47^. A two-photon electron ejection dependence has also been reported from flash photolysis studies of NADH using 355 nm excitation^26,29^. In those studies, the average *I*_*exc*_-levels within the laser excitation pulses were typically more than two orders of magnitude higher than the highest *I*_*exc*_ levels applied in our OPE TRAST measurements (Fig. 1A).

In a similar way as with OPE (Fig. 1C), we recorded TRAST curves of NADH with TPE in the presence of Asc^−^. The recorded TRAST curves (Fig. 3D) also readily reflect the presence of a reducing agent, with decreasing decay amplitudes in the TRAST curves upon Asc^−^ titration, in agreement with an increased rate from **·**NADH^+^ back to ^0^NADH.

#### Global parameter fitting to TRAST curves generated under TPE

TRAST curves recorded under TPE with different *I*_*exc*_ (Fig. 3C) and from samples with different concentrations of Asc^−^ (Fig. 3D) were subject to a global fit, based on a model including electron ejection from a higher excited state, ^n^NADH, as discussed above. The excitation rates were calculated from an NADH TPE cross section of 0.4 GM^48^, and by taking pulse characteristics and spatial distribution of *I*_*exc*_ in the laser focus into account (see Methods and Materials and SI). Under the experimental conditions and range of *w* applied, *k*_*isc*_, *k*_*T*_, *k*_*Q,T*_*(O*_2_) can be expected not to have any significant influence on the TRAST curves and were therefore fixed to the values determined under OPE. ^n^NADH has a very short lifetime ($\ll {\rm{ns}}$) and cannot be resolved on the time-scale of the TRAST experiments. Similar to previous studies of organic fluorophores under saturating OPE^25^, the photo-induced electron ejection can therefore be assumed to take place both via ^1^NADH and ^n^NADH and can be fitted as one effective rate, ${k^{\prime} }_{ee}$. Because of excitation of ^1^NADH to ^n^NADH, ${k^{\prime} }_{ee}$ can also be expected to have an *I*_*exc*_ dependence. The ${k^{\prime} }_{ee}$ rate was therefore fitted individually to each of the TRAST curves in Fig. 3C. The remaining rate parameters, *k*_*red*_, *k*_*deprot*_, *k*_*Q,red*_(Asc^−^), and the diffusion recovery parameter, were all fitted globally, as for the OPE TRAST curves discussed above. The global parameter values, as fitted to the curves in Fig. 3C,D, are ${k}_{red}=2.9\pm 1.4\,{{\rm{\mu }}s}^{-1}$, ${k}_{deprot}=4.4\pm 2.7\,{{\rm{\mu }}s}^{-1}$, and ${k}_{Q,red}({{\rm{Asc}}}^{-})=0.63\pm 0.34\,{{\rm{\mu }}s}^{-1}\,{{\rm{mM}}}^{-1}$. The wider confidence intervals obtained from the TPE data reflect the smaller dataset used and that the maximum speed of the scanner is on the limit to allow analysis of the faster triplet state relaxation of NADH. Nonetheless, all rates are in reasonable agreement with those determined from the OPE TRAST data (Table 1), indicating that they are not influenced by the different mode of excitation (pulsed TPE) and excitation modulation (scanning). The individually fitted ${k^{\prime} }_{ee}$ values are shown in Fig. 3B. Plotted on a log-log scale, they display a slope of 0.9 ± 0.1, suggesting a one photon excitation to occur from ^1^NADH to ^n^NADH, from where a more efficient photo-oxidation then can take place. The slope in Fig. 3B is well in agreement with the *I*_*exc*_-dependence of the overall photo-oxidation rate, as shown in Fig. 3A.

As for the OPE data, we also evaluated alternative photophysical models for the data obtained under TPE, based on the AIC and BIC criteria. Thereby, both a model with an *I*_*exc*_-independent ${k^{\prime} }_{ee}$ rate (two-photon model), and with a ${k^{\prime} }_{ee}$ rate proportional to the photon flux squared (four-photon model) could be clearly discarded (see SI section 8).

### Two-photon NAD(P)H imaging of live cells

#### TRAST imaging

**Figure 4:**
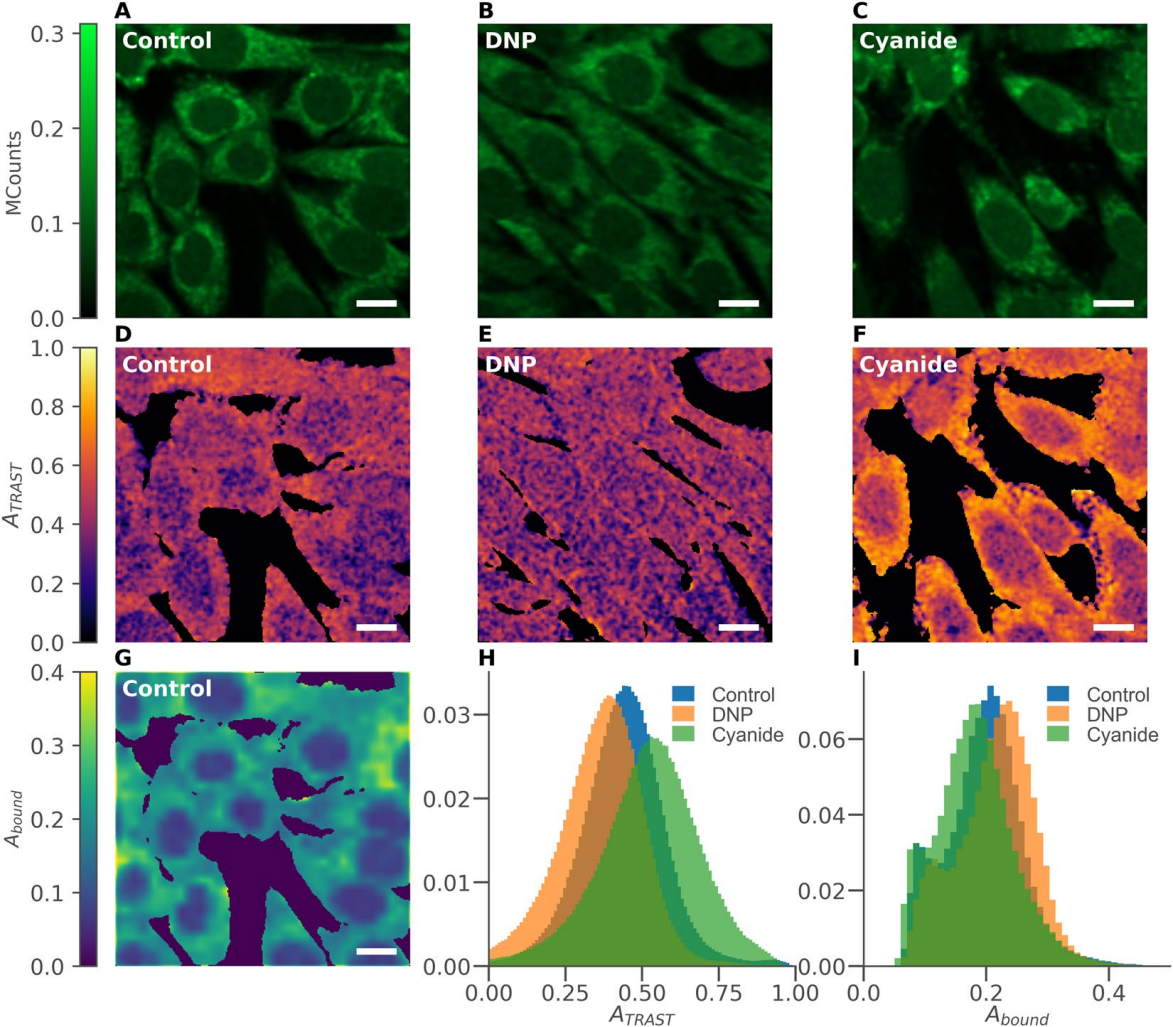
TPE images of cultured mouse myoblast cells in clear DMEM imaging medium (control), or with either 200 μM DNP or 1 mM cyanide added directly to the medium 30 minutes before measurements. Fluorescence intensity images (fast scan), *F*(*w*_*f*_), of (**A**) control cells, (**B**) DNP-exposed cells, and (**C**) cyanide-exposed cells. TRAST images showing the NAD(P)H dark state population, *A*_*TRAST*_ = (*F*(*w*_*f*_) − (*F*(*w*_*s*_))/(*F*(*w*_*f*_), for (**D**) control cells, (**E**) DNP-exposed cells, and (**F**) cyanide-exposed cells, corresponding to the above fluorescence intensity images in A, B, and C. (**G**) Image showing the fraction of bound NAD(P)H, *A*_*bound*_, as determined by FLIM (corresponding to the images in A and D). (**H**) Normalized histograms of *A*_*TRAST*_ in pixels belonging to contol cells (blue), DNP-exposed cells (orange) and cyanide-exposed cells (green). (**I**) Corresponding distributions of *A*_*bound*_ in contol cells (blue), DNP-exposed cells (orange) and cyanide-exposed cells (green). Distributions shown in H and I are based on 131 control cells, 130 DNP-exposed cells, and 160 cyanide-exposed cells. In both histograms, the number of pixles included were: 932494 (control), 917523 (DNP), 1177003 (cyanide). (Scalebars: 10 μm).

We then performed scanned TPE TRAST imaging of unlabelled mouse myoblast cells. Through repeated line-by-line scans over the same field-of-view, while applying different scan speeds, we could extract the same TRAST information as for circular scanning in solution (Fig. 3), but now in a spatially resolved manner (Fig. 4). The photo-damage of cells in TPE microscopy is confined to the focal excitation volume, but follows a non-linear excitation intensity dependence^49^. To minimize photo-toxic effects on the cells, we only used two different scan speeds, with effective durations of excitation faster (*w*_*f*_ = 12 μs) and slower (*w*_*s*_ = 600 μs) than the overall decay time of the TRAST curves, as determined above (Fig. 3). Thereby, the total excitation light dose never exceeded TPE dose levels where long-term (days) photo-toxic effects can start to occur (10 kJ/cm^2^, determined at a peak excitation irradiance of 1.2 TW/cm^2^, twice the one used in this study)^50^. From the difference in average fluorescence intensity, recorded pixel-by-pixel upon fast and slow scanning (*F*(*w*_*f*_) and *F*(*w*_*s*_)), we then imaged the local photo-induced dark state build-up of NADH in the cells as *A*_*TRAST*_ = (*F*(*w*_*f*_) − (*F*(*w*_*s*_))/(*F*(*w*_*f*_). With knowledge of the NADH electronic state model for TPE, this build-up could then be related to changes in the local environment experienced by NAD(P)H in the cells. In parallel with the TRAST imaging, we also recorded full TPE FLIM data from the NAD(P)H fluorescence using time-correlated single photon counting (TCSPC, see Methods and Materials).

Figure 4A shows a regular NAD(P)H fluorescence intensity image, *F*(*w*_*f*_), of unlabelled mouse myoblast cells in a clear DMEM imaging medium. The corresponding TRAST image is shown in Fig. 4D, displaying a lower NAD(P)H dark state population in the cell nuclei as opposed to the cytosol. Averaging *A*_*TRAST*_ over 131 cells confirmed this observation and resulted in an average dark state population of 〈*A*_*TRAST*_〉_*control*_ = 0.43 ± 0.02, with a clear separation between the nuclear, 〈*A*_*TRAST*_〉_*nucl*_ = 0.38 ± 0.02, and cytosolic, 〈*A*_*TRAST*_〉_*cyto*_ = 0.45 ± 0.02, regions.

To investigate how changes in the metabolic states of the cells can influence *A*_*TRAST*_ of NAD(P)H, we also imaged cells with either 1 mM cyanide (n = 160 cells) or 200 μM of the mitochondrial un-coupler dinitrophenol (DNP)^51^ (n = 130) added to the culturing medium 30 minutes before acquiring the images (see Methods and Materials). While no particular differences can be identified in the fluorescence intensity images (Fig. 4A–C), the TRAST images revealed a significant increase in *A*_*TRAST*_ of NAD(P)H in cells exposed to cyanide (Fig. 4F), with 〈*A*_*TRAST*_〉_*cyanide*_ = 0.52 ± 0.02. In contrast, cells exposed to DNP showed a decreased dark state amplitude, 〈*A*_*TRAST*_〉_*DNP*_ = 0.36 ± 0.02, particularly in the cytosolic regions. The opposite effects of DNP and cyanide exposure on *A*_*TRAST*_ likely reflect the opposite effects of these compounds on the electron transport chain (ETC) in the mitochondria of the cells. While DNP as an un-coupler induces an increased activity in the ETC, cyanide is an ETC inhibitor^11^. Histograms of *A*_*TRAST*_ values from these three different categories of cells (control, DNP-exposed, cyanide-exposed), recorded from individual pixels in the TRAST images, are shown in Fig. 4H. The spread in *A*_*TRAST*_ values largely reflects differences in the oxidative environment between nuclear and cytosolic regions of the cells, in particular for the cyanide exposed cells. Despite this spread however, the different categories of cells can be clearly distinguished based on these whole cell pixel histograms.

TPE scanned TRAST measurements of NADH in TRIS buffer showed no effect of either 1 mM cyanide or 200 μM DNP (data not shown), i.e. cyanide and DNP themselves did not directly influence *A*_*TRAST*_.

#### TRAST imaging, combined with fluorescence lifetime data

Next, we analysed how the TRAST images, as recorded above using TCSPC, relates to FLIM-analysis of the same data. The combined TCSPC data from all measured cells was fitted to a two-exponential fluorescence decay model (see Methods and Materials). The two lifetimes, representing the average lifetimes of free and bound NAD(P)H in the cells, were determined to *τ*_*free*_ = 0.4 ns and *τ*_*bound*_ = 2.4 ns. The *τ*_*free*_ value is well in agreement with what has been reported previously^12,52^. *τ*_*bound*_ depends on what proteins NAD(P)H is bound to, as well as on local viscosity. Our average *τ*_*bound*_ value lies well within the reported range of fluorescence lifetimes of protein-bound NAD(P)H (around 1 ns up to 3.5 ns), in solution or in different cells^3,12,17,52,53^. It also lies in between reported intracellular lifetimes of NADH (~1.5 ns) and NADPH (~4 ns)^54^. With *τ*_*free*_ and *τ*_*bound*_ fixed to 0.4 ns and 2.4 ns, respectively, the relative amplitudes of the two lifetime components were fitted pixel-wise in the cellular images, using a maximum likelihood estimator (MLE) (see Methods and Materials). Correcting for the difference in fluorescence brightness (by assuming that the fluorescence quantum yields of the free and bound fractions are proportional to *τ*_*free*_ and *τ*_*bound*_, respectively) allows the bound fraction of NAD(P)H, *A*_*bound*_, to be calculated in each pixel. Thereby, cellular images showing the fraction of bound NAD(P)H were generated (Fig. 4G), revealing a lower *A*_*bound*_ in the nuclear regions of the cells. The cytosol regions of the cells were found to contain sub-regions with enhanced levels of bound NAD(P)H, localized around the nuclei. Their locations corresponds to that expected for mitochondria, in which typically also a larger fraction of bound NAD(P)H is found^18,54^. However, when comparing *A*_*bound*_ between control cells and those exposed to cyanide or DNP, only minor differences could be observed (Fig. 4I), with 〈*A*_*bound*_〉_*control*_ = 0.20 ± 0.01, 〈*A*_*bound*_〉_*cyanide*_ = 0.18 ± 0.01, and 〈*A*_*bound*_〉_*DNP*_ = 0.21 ± 0.01. The effects observed in the FLIM data upon addition of DNP are minor, and not as evident as the differences we see in the dark state population by TRAST imaging (Fig. 4H). Similarly, the effects observed upon addition of cyanide are more prominent in the TRAST compared to the FLIM images. Like 〈*A*_*TRAST*_〉, also 〈*A*_*bound*_〉 shows opposite effects upon addition of DNP and cyanide, reflecting the different mechanisms of action of these compounds. The slight increase and decrease in *A*_*bound*_ found in cells exposed to DNP and cyanide, respectively, is in agreement with previous NAD(P)H FLIM studies of cells exposed to similar uncouplers and blockers, when added over comparable exposure times and doses^18,19,54^.

**Figure 5:**
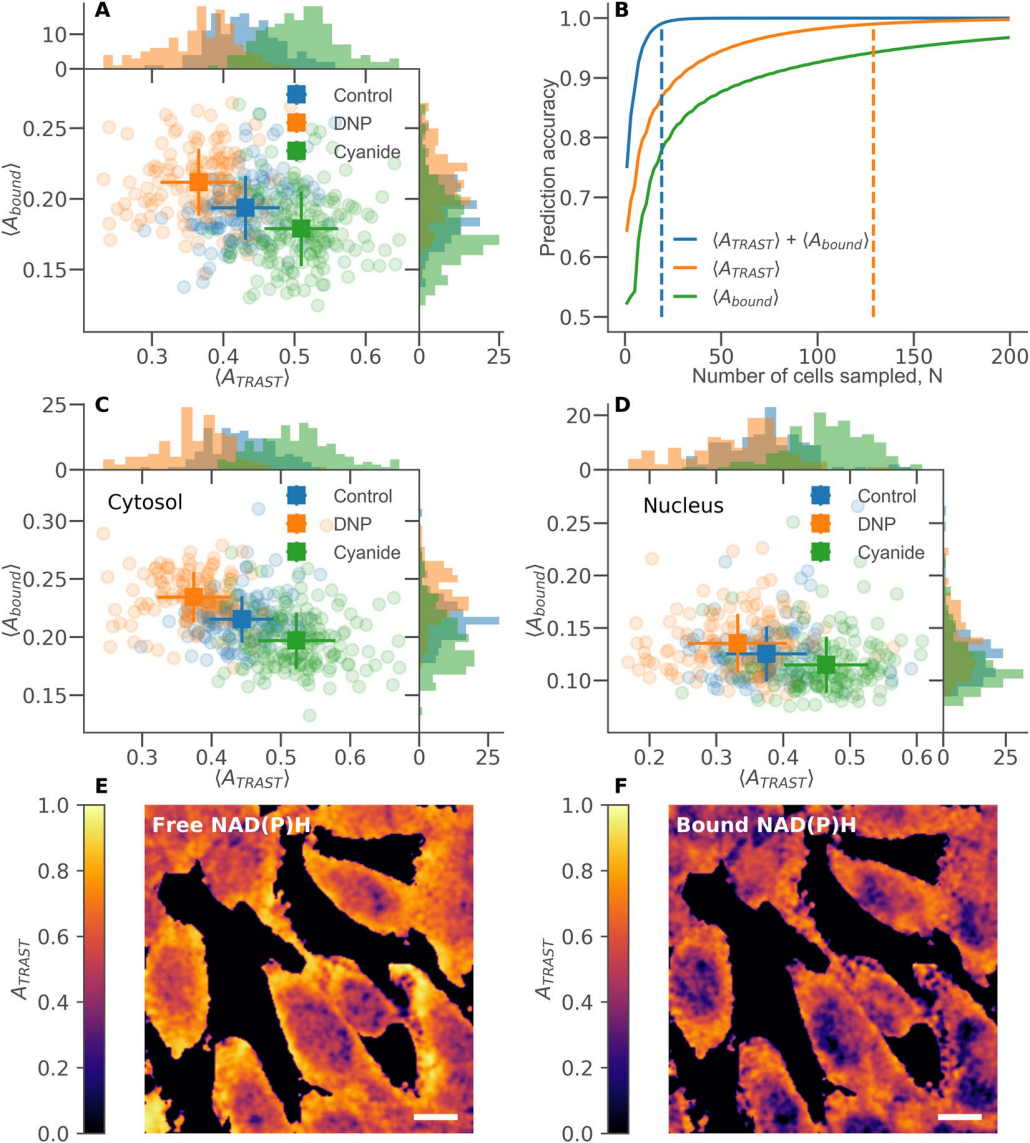
(**A**) Distributions of average dark state amplitudes, 〈*A*_*TRAST*_〉, and bound fractions, 〈*A*_*bound*_〉, of NAD(P)H, calculated as individual cell averages for control cells and cells exposed to either 200 μM DNP or 1 mM cyanide. Square markers indicate sample average and standard deviation. (**B**) Average prediction accuracy as a function of number of cells sampled, N, when classifying cells as belonging to either the control, DNP-exposed or cyanide-exposed group. The classification was based on the machine learning procedure described in Methods and Materials. Utilizing both 〈*A*_*TRAST*_〉 and 〈*A*_*bound*_〉 together results in the highest accuracy. Dashed lines indicate the point of 99% accuracy (19 cells for *A*_*TRAST*_ combined with 〈*A*_*bound*_〉, 129 cells for 〈*A*_*TRAST*_〉 only, ≫200 for 〈*A*_*bound*_〉 only). Manually selecting the cell nuclei in each image allows the separation of Fig. 5A into the distribution for the cytosolic (**C**) and nuclear regions (**D**). Utilizing the images of *A*_*bound*_ (e.g. Fig. 4G) for both fast and slow scanning allows TRAST images to be generated for free (**E**) and bound (**F**) NAD(P)H separately. Both these images are extracted from the image in Fig. 4F, showing cyanide-exposed cells. (Scalebars: 10 μm).

Overall Fig. 4A–I thus indicate that the NAD(P)H dark state population, as imaged by TRAST, can reflect changes in the local environment upon addition of either cyanide or DNP. These changes are also reflected in the NAD(P)H bound fraction, as imaged by FLIM, although to a smaller degree. To investigate to what extent both TRAST and lifetime data can be used to identify cells based on such changes, we calculated the average bound fraction, 〈*A*_*bound*_〉, and dark state amplitude, 〈*A*_*TRAST*_〉, for each individual cell studied above, see Fig. 5A. We then used a standard machine learning algorithm (*random forest classifier, scikit-learn, python*, see Method and Materials) to perform a simple classification of all the 421 cells studied as belonging to either the control, DNP-exposed, or cyanide-exposed group. On an individual cell level, the average classification accuracy when using 〈*A*_*TRAST*_〉 was 64%, while the corresponding analysis based on 〈*A*_*bound*_〉 resulted in 52% of the cells being correctly identified. Using both 〈*A*_*TRAST*_〉 and 〈*A*_*bound*_〉 together resulted in a better classification accuracy of 75% for single cells, indicating that the two parameters represent at least partially independent information. All single cell classification probabilities are summarized in Supplementary Fig. S5. The predictive capacity can be considerably improved by sampling more than one cell from the same category. Figure 5B shows the extrapolated accuracies, based on the probabilities in Supplementary Fig. S5, to correctly classify a group of *N* cells. In this case each individual cell is evaluated separately, and the final category is assigned based on the most common result. In our case, a 99% accuracy would be obtained if both TRAST and lifetime data from 19 cells is used. The same accuracy using only TRAST data requires 129 cells, while using only lifetime data would require $\gg 200$ cells.

As seen in Fig. 4D–F, additions of DNP or cyanide tend to affect *A*_*TRAST*_ differently in nuclear and cytosolic regions of the cells. With the stronger response generally seen in the cytosolic regions, the classification accuracy could potentially be increased if only pixels from the cytosol are included. In Fig. 5C,D, the corresponding single cell averages as in Fig. 5A are shown, but now only including pixels from the cytosolic and nuclear regions of the cells, respectively. In our case, only a minor benefit in the classification could be noted.

The general trend observed in Fig. 5A is that there is a negative correlation between 〈*A*_*TRAST*_〉 and 〈*A*_*bound*_〉 upon perturbation of the cells with either DNP or cyanide. This observation might seem counter-intuitive from a photodynamical point of view (Fig. 2), since an increase in 〈*A*_*bound*_〉 also represents an increase in the average NAD(P)H fluorescence lifetime, 〈*τ*_*F*_〉. In a model where excitation to ^1^NADH (or ^n^NADH) leads to dark state formation with an average electron ejection quantum yield of $\langle {\Phi }_{ee}\rangle ={k}_{ee}\cdot \langle {\tau }_{F}\rangle$, an increased fluorescence lifetime is expected to result in a correspondingly higher photo-induced dark state formation. However, flash photolysis studies of NADH^27^ indicate that electron ejection, observed via transient absorption of solvated electrons, is not directly coupled to the excitation (since the absorption is not observed within the time of the laser excitation pulse) or to the population of ^1^NADH (since the transient absorption of solvated electrons appears at a much faster time-scale than the lifetime of ^1^NADH). Electron ejection was instead suggested to take place from an “intermediate state”, possibly an excited vibrational state of ^1^NADH. If electron ejection does not take place from a vibrationally relaxed ^1^NADH, then the electron ejection yield can be independent of *τ*_*F*_. This can explain the observation that 〈*A*_*TRAST*_〉 and 〈*A*_*bound*_〉 are not directly coupled, and suggests that 〈*A*_*TRAST*_〉 does indeed reflect a changed oxidative environment in the cell, as opposed to the variations in fluorescence lifetime (bound fraction) reported by FLIM. This implies that FLIM and TRAST imaging data do provide orthogonal information and that better identification of cellular metabolic states is possible when both modalities are used in parallel.

Given that TRAST and FLIM data is imaged in parallel, it is also possible to produce TRAST images based on subsets of the recorded photon counts. Using the fractions of bound NAD(P)H, determined by FLIM in both the fast and slow fluorescence images, it is possible to calculate *A*_*TRAST*_ for free and bound NAD(P)H separately. This is demonstrated in Fig. 5E,F, where a TRAST image of cyanide-exposed cells (Fig. 4F) has been separated into the contributions from free and bound NAD(P)H.

In conclusion, we show in this work that the dynamics of photo-induced dark states of NAD(P)H can be monitored by TRAST. The transient state data obtained by TRAST compares well with flash photolysis data but can be applied on a much broader range of samples and measurement conditions. We show that TRAST imaging of NAD(P)H can be applied on live cells, adds information about the local metabolic state of the cells, and allows classification of cells with higher accuracy than when based on FLIM alone. TRAST imaging is straight-forward and can be applied in parallel with FLIM for label-free cellular metabolic imaging. This will add additional independent parameters, increase specificity and sensitivity and thereby help overcome some of the limitations encountered in such measurements.

## Methods and Materials

### Theory of transient state (TRAST) spectroscopy

TRAST spectroscopy determines fluorophore blinking kinetics by monitoring how systematic changes in the excitation pattern affect the time-averaged fluorescence signal returned^30–33,55,56^. In this work, we implemented TRAST in a confocal setting, first using on/off modulation of the stationary excitation laser, and later in a scanned configuration by systematically varying the scan speed whilst imaging.

#### Stationary TRAST

The emitted luminescence intensity from a sample can for most fluorophores, including NADH, be considered proportional to the population of the first excited singlet state. The time averaged fluorescence signal resulting from a rectangular excitation pulse of duration *w* is then

1 $$\begin{array}{c}\langle {F}_{exc}(w)\rangle =c{q}_{f}{q}_{D}{k}_{10}\frac{1}{w}{\int }_{t=0}^{w}(\iiint {}^{1}NADH(\bar{r},t)\,CEF(\bar{r})\,dV)\,dt\end{array}$$

where $CEF(\bar{r})$ is the normalized collection efficiency function, *c* is the fluorophore concentration, *q*_*f*_ is the fluorescence quantum yield and *q*_*D*_ is the overall detection quantum yield of the instrument. The simulations of the excited singlet state population, ${}^{1}NADH(\bar{r},t)$, are outlined in SI section 1, with further simplifications to Eq. (1) described in Data Analysis and in SI sections 4 and 5. By monitoring the change in 〈*F*_*exc*_(*w*)〉 in response to varying pulse durations, the set of photophysical rate parameters that best match the experimental data can be determined (see Data Analysis).

In order to collect enough photons even for short *w*, pulse trains consisting of *N* identical excitation pulses, of pulse period *T*, are recorded onto a single exposure of the integrating detector. For each pulse train, a constant illumination time, ${t}_{ill}=N\cdot w$, is typically maintained. At the same time, the excitation duty cycle, *η* = *w/T*, is kept low to make sure the sample can relax back to the same initial condition before the onset of the next excitation pulse. Each value of *w* then completely defines an excitation pulse train, recorded in its entirety by the detector using an exposure time of ${t}_{exp}={t}_{ill}/\eta$. A so-called TRAST curve is produced by measuring 〈*F*_*exc*_(*w*)〉 from pulse trains over a range of excitation durations and normalizing at the shortest pulse duration used, *w*_0_.

2 $$\begin{array}{c}{\langle {F}_{exc}(w)\rangle }_{norm}=(\frac{1}{N}\mathop{\sum }\limits_{i=1}^{N}{\langle {F}_{exc}(w)\rangle }_{i})/(\frac{1}{{N}_{0}}\mathop{\sum }\limits_{i=1}^{{N}_{0}}{\langle {F}_{exc}({w}_{0})\rangle }_{i})\end{array}$$

where 〈*F*_*exc*_(*w*)〉_*i*_ is the fluorescence response from the *i*:th pulse in the pulse train. If the duty cycle is low, 〈*F*_*exc*_(*w*)〉_*i*_ can be assumed identical for all *i* and the expression simplifies further. Note that the normalization step of Eq. (2) cancels out many experimental constants in the calculation of 〈*F*_*exc*_(*w*)〉_*i*_, e.g. fluorophore quantum yield, sample concentration and overall detection efficiency of the optical system.

#### Scanned TRAST with TPE

Excitation modulation can also be achieved by scanning a focused excitation beam across the sample, using the scan speed, *v*, to control the duration of excitation, *w*. For a homogeneous solution sample we get

3 $$\begin{array}{c}\langle {F}_{exc}(v)\rangle =c{q}_{f}{q}_{D}{k}_{10}\iiint {}^{1}N{\rm{ADH}}(\bar{r},v)\cdot CEF(\bar{r})\,dV\end{array}$$

Here the coordinates are defined from the perspective of the laser, such that the normalized $CEF(\bar{r})$ remains time-independent. A TRAST curve is then produced by converting the scan speed to an effective fluorophore excitation duration, *w*_*eff*_ (see SI section 3), and then, analogous to Eq. (2), normalizing by the excitation duration corresponding to the fastest scan speed, *w*_*eff*,0_

4 $${\langle {F}_{exc}({w}_{eff})\rangle }_{norm}=\langle {F}_{exc}({w}_{eff})\rangle /\langle {F}_{exc}({w}_{eff,0})\rangle $$

**Figure 6:**
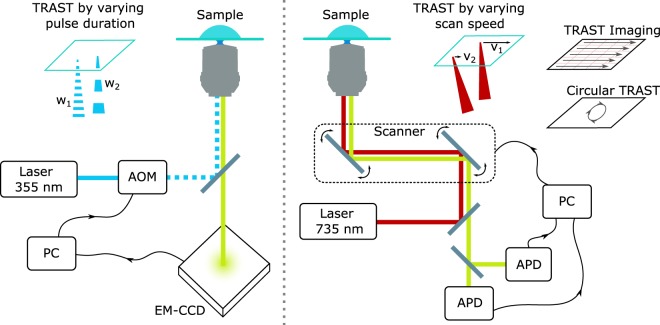
Schematic representation of stationary TRAST (**A**) and scanned TRAST (**B**). See Instrumentation for details.

If TRAST imaging is to be performed, the fluorescence time trace is first binned to pixels before the TRAST ratio is taken for each pixel separately. This TRAST approach can be realized on any standard confocal scanning microscope, without the need for intensity modulation (see Fig. 6). Furthermore, measurement times can be significantly reduced compared to stationary TRAST, since photon collection does not have to be interrupted to allow the sample to recover. Suitable fluorophore recovery time between excitations is instead achieved by setting the length of the path to be scanned, while photon collection is continuously ongoing at other locations in the sample. Supplementary Fig. S3 shows an example of an intensity trace recorded using scanned TRAST in NADH solution.

Compared to stationary TRAST, simulating the speed dependent ${}^{1}N{\rm{ADH}}(\bar{r},v)$ is much more computationally intensive. The excitation pulses in scanned TRAST are no longer rectangular in time since scanning with a Gaussian beam creates a continuous rise and fall of the excitation rate, *k*_01_(*t*), as the beam passes by. The TPE laser is also pulsed, adding a fs time dependence to the excitation pattern as well. In this work, we designed a simplified model with continuous rectangular excitation pulses of amplitudes and durations such as to generate equivalent effects on the NADH transient state build-up. The fs pulse micro-time averaging is described in SI section 2 and the macro-time variations due to scanner movement in SI section 3.

### Instrumentation

#### OPE TRAST measurements

One-photon measurements were performed on a home built, epi-illuminated microscope using a 355 nm continuous wave  (CW) solid state laser (Cobolt Zouk, 20 mW) for excitation. The laser beam was chopped to square pulses by an acousto-optical modulator (AOM; MQ110-A1-UV, AA Opto-Electronics, Orsay), and then focused into the sample by a water immersion objective (40×, NA 1.2, Zeiss, C-Apochromat). The 1/e^2^-radius of the laser beam in the focal plane was 863 nm. Fluorescence from the sample was collected through the same objective, separated from the excitation light by a dichroic beamsplitter (Di02-R405, Semrock) and a 364 nm long-pass filter (BL01–364R, Semrock), and then detected by a 658 × 496 pixel EM-CCD camera (Andor Luca). By only considering the signal focused on a few central pixels, an effective 100 μm pinhole was introduced. Control of the AOM modulation as well as synchronisation with the detection was handled by a digital I/O card (PCI-6602, National Instruments) and a custom Matlab script.

Excitation pulse durations, *w*, were set from 500 ns to 1 ms and were distributed logarithmically. For a given *w*, the number of identical pulse repetitions, *N*, was selected to maintain a constant total illumination time, *t*_*ill*−*OPE*_ = *w* · *N* = 50 ms. The pulse train duty cycle was set to *η* =1%. Any bleaching of the sample was accounted for by inserting brightness reference measurements as every third pulse train. For these reference measurements, we used the shortest available pulse duration, *w*_0_, to avoid any dark state build-up.

#### TPE TRAST measurements

The measurements were based on a commercial, epi-illuminated, confocal laser scanning microscope (Olympus FV1200) with a water immersion objective (60×, NA 1.2, Olympus, UPlanSApo) and the pinhole set to its maximum size (800 μm). The microscope was modified to allow external control of the galvo-scanning mirrors using a digital I/O card (DaqBoard/3001USB, Measurement Computing) controlled by custom software written in Matlab. The excitation source was a mode-locked Ti:Sapphire laser (Coherent, Mira) tuned to 735 nm, with a repetition rate of 76 MHz and a pulse width measured to 150 fs FWHM. The beam radius in the focal plane was measured to 327 nm (1/e^2^) by scanning 24 nm fluorescent beads (FluoSpheres, Carboxylate-Modified Microspheres 505/515, #F8787, Invitrogen) deposited on a regular cover slide. Fluorescence light was separated from the excitation light by a 680 nm dichroic mirror (Chroma) and a 680 nm short-pass filter (ET680sp-2P8, Chroma), before focused onto two APD detectors (Perkin & Elmer, SPCM-AQR-14) connected to a TCSPC module (HydraHarp 400). An additional emission filter (HQ445/60, Chroma) was added for cell-imaging to separate the NAD(P)H emission from that of other autofluorescent species in the cells.

For solution measurements, a circular scanning pattern of 28.3 μm diameter was used, with the scan speed adjusted in 14 steps to yield effective excitation pulse durations between 2 μs and 690 μs (see SI section 3). Data was collected for 0.5 s at each fixed speed, and the whole measurement sequence was repeated 20 times for a total illumination time of *t*_*ill*−*TPE*_ = 20 · 0.5s = 10s per scan speed. Any sample bleaching or evaporation could be tracked directly in the data, since each scan speed was repeated 20 times (see Supplementary Fig. S3 for an example of scanned TRAST data).

For live cell scanned TRAST imaging, traditional line-by-line scanning was performed in a 90 × 90 μm field of view, binned to 128 × 128 pixels. The excitation intensity was measured to 2.6 MW/cm^2^. To minimize photo-toxicity, only two scan speeds (corresponding to *w*_*f*_ = 12 μs and *w*_*s*_ = 600 μs effective laser dwell times) were used. The faster scan was repeated 50 times in order to reach directly comparable photon counts, i.e. 50*w*_*f*_ = *w*_*s*_. These repeated frames also serve as bleaching reference points for each pixel. After image alignment (see Data Analysis), the pixel-by-pixel TRAST decay amplitude was extracted as the normalized difference between the two images, *A*_*TRAST*_ = (*F*(*w*_*f*_) − (*F*(*w*_*s*_))/(*F*(*w*_*f*_).

### Data analysis

#### Solution measurements

TRAST curves were analysed using custom software implemented in Matlab. Pre-processing included the subtraction of a static background component as well as corrections for any changes in sample concentration due to bleaching or evaporation. The background is due to ambient light and detector dark counts and typically amounted to 100 counts per second and pixel, corresponding to about 10% of the total signal in our measurements. The concentration correction was based on brightness references acquired throughout each measurement at regular intervals. The effect of bleaching turned out to be negligible, while concentration changes due to evaporation typically caused around 2% drift in brightness over the course of one measurement.

Stationary TRAST curves, recorded under OPE with a stationary laser beam, were simulated by use of eq. (S3), using the average excitation rate in eq. (S12) (see SI section 5) and the simplified diffusion model in eq. (S10) (see SI section 4). For a given photophysical model, the rate parameters that best described the experimental data were then found through non-linear least squares optimization.

For TRAST data acquired by laser scanning, we used a rectangular pulse shape approximation described in SI section 3 as well as the TPE pulse averaging in eq. (S6) (SI section 2) and spatial averaging in eq. (S13) in SI section 5. Thereby, scanned TRAST data could be fitted in the same way as stationary TRAST curves, with no modifications to the fitting algorithm.

#### Scanned TRAST Imaging

Scanned TRAST images were expressed as the pixel-by-pixel dark state amplitude, calculated by comparing the recorded average fluorescence intensity from fast and slow scanning, *A*_*TRAST*_ = (*F*(*w*_*f*_) − (*F*(*w*_*s*_))/(*F*(*w*_*f*_).

When recording confocal laser scanning images, a scan-speed dependent offset of the images was observed. The difference between fast and slow scanning amounted to 0.63 μm or a fixed 0.9 pixel offset. This offset becomes crucial if ratio images are to be produced and an image alignment step was added to prevent edge effect near high contrast regions of the images. First, both images were oversampled (using scikit-image, v. 0.21.2, in Python) by a factor of 10 and translated to maximize the correlation between the two. The overlaid images were then returned to original resolution before calculating a TRAST image. This procedure is very similar to the treatment in^31^.

#### FLIM and free/bound NADH ratios

The data acquired for scanned TRAST imaging, could also be used for FLIM analysis. To get the best possible photon statistics, the combined dataset of all measured cells was first fitted to two lifetime components, yielding *τ*_*free*_ = 0.4 ns and an average *τ*_*bound*_ = 2.4 ns (see Results). These lifetimes were then fixed, and only their relative amplitude was fitted for each pixel individually using maximum likelihood estimation (MLE). The MLE included deconvolution with the instrument response function (IRF) for each respective detection channel and the estimated amplitude was weighted by relative brightness to obtain the fraction of bound NAD(P)H in each pixel (Fig. 4G). With the large difference between *τ*_*free*_ and *τ*_*bound*_, the MLE could reliably differentiate between the short and long lifetime components, even if some uncertainty in the local lifetime of bound NAD(P)H is assumed. The estimated bound fraction showed only minor variations when *τ*_*bound*_ was varied between 2 and 3 ns.

#### Cell classification using machine learning

The boundary of each cell (in total 131 control, 130 DNP-exposed, and 160 cyanide-exposed cells) was manually selected in the fluorescence intensity images (e.g. Fig. 4A). For each cell, the average *A*_*TRAST*_ and bound fraction of NAD(P)H was then calculated based on the corresponding TRAST (e.g. Fig. 4D) and lifetime (e.g. Fig. 4G) images. A standard python machine learning algorithm (*scikit-learn, v. 0.21.2 sklearn.ensemble.RandomForestClassifier*) was used with default parameters, except for *n_estimators* = 500 and *max_depth* = 4. Training was performed on a random subset consisting of 80% of the cells, while the remaining 20% were kept for evaluating the model’s predictive capacity. Given the small sample set (421 cells) we report the average prediction accuracy based on 10^3^ training/evaluation runs, each using a new random subset of cells for training. The resulting probability matrices for cell classification are shown in Supplementary Fig. S5. These matrices were then used to calculate the prediction accuracy when evaluating multiple cells of the same type, as seen in Fig. 5B. This combined predictive accuracy refers to the total probability of all possible combinations of individual cell classification which result in the correct category receiving more votes than either of the two incorrect alternatives. In the classification in Fig. 5B, groups of control, DNP-exposed and cyanide-exposed cells were assumed to be equally likely to occur.

### Sample preparation

Stocks of β-nicotinamide adenine dinucleotide (NADH; reduced disodium salt, Sigma N0786), β-nicotinamide adenine dinucleotide 2′-phosphate (NADPH; reduced tetrasodium salt, Sigma N0411), as well as TRIS buffer, sodium ascorbate, hydrogen peroxide and sodium hydroxide were all purchased from Sigma. Aliquots of 1 mM NADH in 50 mM TRIS buffer (pH 7.4) were prepared and stored at −80 °C. A new vial was thawed on every measurement day and further diluted using the same TRIS buffer. NADPH was treated in the same way. Solutions of sodium ascorbate were prepared daily using the same TRIS buffer.

Experiments with 355 nm excitation used quartz cover slides (CFQ-2417, UQG Optics) to avoid the strong background seen from regular glass cover slips at this wavelength.

Experiments with modified atmospheres were performed in a lightly pressurized chamber (custom built, 14 ml cylindrical container, bottom made from a replaceable CFQ-2417 quartz cover slide) with a constant flow of either argon or oxygen. The gas first flowed through a bubble humidifier to reduce evaporation of the sample.

### Cell culture

C_2_C_12_ cells (mouse myoblasts, ATCC CRL-1772) were grown in Gibco DMEM/F-12 (1:1) + GlutaMAX medium (ThermoFisher, 31331–028) supplemented by 10% FBS (Biowest, VWR #S1810–500). 24 hours before measurements, cells were seeded in #1.5 glass bottom microscopy wells (Nunc Lab-Tek II 8-well Chambered Coverglass), using the same medium. 30 minutes before measurements, the medium was changed to clear Gibco FluoroBrite DMEM imaging medium (ThermoFisher, A18967–01). The imaging medium contained either no additions (“control cells”), 200 µM dinitrophenol (Sigma D198501) (“DNP-exposed cells”) or 1 mM potassium cyanide (Sigma 60178) (“cyanide-exposed cells”).

## Supplementary information

Supplementary Information

## Data Availability

The datasets generated during and/or analysed during the current study are available from the corresponding author on reasonable request.

## References

[1] Srivastava S (2016). Emerging therapeutic roles for NAD(+) metabolism in mitochondrial and age-related disorders. Clinical and Translational Medicine.

[2] Martinez-Outschoorn UE, Peiris-Pages M, Pestell RG, Sotgia F, Lisanti MP (2017). Cancer metabolism: a therapeutic perspective. Nature Reviews Clinical Oncology.

[3] Chakraborty S, Nian FS, Tsai JW, Karmenyan A, Chiou A (2016). Quantification of the Metabolic State in Cell-Model of Parkinson’s Disease by Fluorescence Lifetime Imaging Microscopy. Scientific Reports.

[4] Ying WH (2008). NAD(+)/ NADH and NADP(+)/NADPH in cellular functions and cell death: Regulation and biological consequences. Antioxidants & Redox Signaling.

[5] Denk W, Strickler JH, Webb WW (1990). 2-photon laser scanning fluorescence microscopy. Science.

[6] Huang SH, Heikal AA, Webb WW (2002). Two-photon fluorescence spectroscopy and microscopy of NAD(P)H and flavoprotein. Biophysical Journal.

[7] Skala MC (2007). *In vivo* multiphoton microscopy of NADH and FAD redox states, fluorescence lifetimes, and cellular morphology in precancerous epithelia. Proc. Natl. Acad. Sci. USA.

[8] Kolenc OI, Quinn KP (2019). Evaluating Cell Metabolism Through Autofluorescence Imaging of NAD(P)H and FAD. Antioxidants & Redox Signaling.

[9] Chance B, Schoener B, Oshino R, Itshak F, Nakase Y (1979). Oxidation-reduction ratio studies of mitochondria in freeze-trapped samples - NADH and flavoprotein fluorescence signals. Journal of Biological Chemistry.

[10] Hou J (2016). Correlating two-photon excited fluorescence imaging of breast cancer cellular redox state with seahorse flux analysis of normalized cellular oxygen consumption. Journal of Biomedical Optics.

[11] Blacker TS, Duchen MR (2016). Investigating mitochondrial redox state using NADH and NADPH autofluorescence. Free Radical Biology and Medicine.

[12] Lakowicz JR, Szmacinski H, Nowaczyk K, Johnson ML (1992). Fluorescence lifetime imaging of free and protein-bound NADH. Proc. Natl. Acad. Sci. USA.

[13] Chorvat D, Chorvatova A (2009). Multi-wavelength fluorescence lifetime spectroscopy: a new approach to the study of endogenous fluorescence in living cells and tissues. Laser Physics Letters.

[14] Yu QR, Heikal AA (2009). Two-photon autofluorescence dynamics imaging reveals sensitivity of intracellular NADH concentration and conformation to cell physiology at the single-cell level. Journal of Photochemistry and Photobiology B-Biology.

[15] Skala MC (2007). *In vivo* multiphoton fluorescence lifetime imaging of protein-bound and free nicotinamide adenine dinucleotide in normal and precancerous epithelia. Journal of Biomedical Optics.

[16] Wang HW (2008). Differentiation of apoptosis from necrosis by dynamic changes of reduced nicotinamide adenine dinucleotide fluorescence lifetime in live cells. Journal of Biomedical Optics.

[17] Plotegher N (2015). NADH fluorescence lifetime is an endogenous reporter of alpha-synuclein aggregation in live cells. FASEB Journal.

[18] Li D, Zheng W, Qu JY (2008). Time-resolved spectroscopic imaging reveals the fundamentals of cellular NADH fluorescence. Optics Letters.

[19] Kalinina S (2016). Correlative NAD(P)H-FLIM and oxygen sensing-PLIM for metabolic mapping. Journal of Biophotonics.

[20] Dravid UA, Mazumder N (2018). Types of advanced optical microscopy techniques for breast cancer research: a review. Lasers in Medical Science.

[21] Scott TG, Spencer RD, Leonard NJ, Weber G (1970). Emission properties of NADH. Studeis of fluorescence lifetimes and quantum efficiencies of NADH, AcPyADH, and simplified synthetic models. Journal of the American Chemical Society.

[22] Visser A, Vanhoek A (1981). The fluorescence decay of reduced nicotinamides in aqueous-solution after excitation with a UV-mode locked Ar ion laser. Photochemistry and Photobiology.

[23] Drozdowicz-Tomsia K (2014). Multiphoton fluorescence lifetime imaging microscopy reveals free-to-bound NADH ratio changes associated with metabolic inhibition. Journal of Biomedical Optics.

[24] Widengren J, Mets Ü, Rigler R (1995). Fluorescence correlation spectroscopy of triplet-states in solution - a theoretical and experimental-study. Journal of Physical Chemistry.

[25] Widengren J, Chmyrov A, Eggeling C, Löfdahl PÅ, Seidel CAM (2007). Strategies to improve photostabilities in ultrasensitive fluorescence spectroscopy. Journal of Physical Chemistry A.

[26] Lindqvist L, Czochralska B, Grigorov I (1985). Determination of the mechanism of photo-ionization of NADH in aqueous-solution on laser excitation at 355 nm. Chemical Physics Letters.

[27] Boldridge DW, Morton TH, Scott GW (1984). Formation kinetics and quantum yield of photon-induced electron ejection from NADH in aqueous-solution. Chemical Physics Letters.

[28] Umrikhina AV, Luganskaya AN, Krasnovsky AA (1990). ESR signals of NADH and NADPH under illumination. FEBS Letters.

[29] Czochralska B, Lindqvist L (1983). Biphotonic one-electron oxidation of NADH on laser excitation at 353 nm. Chemical Physics Letters.

[30] Sandén T, Persson G, Thyberg P, Blom H, Widengren J (2007). Monitoring kinetics of highly environment sensitive states of fluorescent molecules by modulated excitation and time-averaged fluorescence intensity recording. Analytical Chemistry.

[31] Sandén T, Persson G, Widengren J (2008). Transient State Imaging for Microenvironmental Monitoring by Laser Scanning Microscopy. Analytical Chemistry.

[32] Hevekerl H, Tornmalm J, Widengren J (2016). Fluorescence-based characterization of non-fluorescent transient states of tryptophan - prospects for protein conformation and interaction studies. Scientific Reports.

[33] Tornmalm J, Widengren J (2018). Label-free monitoring of ambient oxygenation and redox conditions using the photodynamics of flavin compounds and transient state (TRAST) spectroscopy. Methods.

[34] Ross JBA, Rousslang KW, Motten AG, Kwiram AL (1979). Base interactions in the triplet state of NAD+ and NADH. Biochemistry.

[35] Nikandrov VV, Brin GP, Krasnovskii AA (1978). Light-induced activation of NADH and NADPH. Biochemistry-Moscow.

[36] Fukuzumi S, Inada O, Suenobu T (2002). Direct detection of radical cations of NADH analogues. Journal of the American Chemical Society.

[37] Chmyrov A, Sandén T, Widengren J (2010). Iodide as a Fluorescence Quencher and Promoter-Mechanisms and Possible Implications. Journal of Physical Chemistry B.

[38] Anne A, Hapiot P, Moiroux J, Neta P, Saveant JM (1992). Dynamics of proton-transfer from cation radicals - kinetic and thermodynamic acidities of cation radicals of nadh analogs. Journal of the American Chemical Society.

[39] Gebicki J, Marcinek A, Zielonka J (2004). Transient species in the stepwise interconversion of NADH and NAD(+). Accounts of Chemical Research.

[40] Zielonka J, Marcinek A, Adamus J, Gebicki J (2003). Direct observation of NADH radical cation generated in reactions with one-electron oxidants. Journal of Physical Chemistry A.

[41] Czochralska B, Kawczynski W, Bartosz G, Shugar D (1984). Oxidation of excited-state NADH and NAD dimer in aqueous-medium involvement of O_2−_ as a mediator in the presence of oxygen. Biochimica Et Biophysica Acta.

[42] Akaike H (1974). A new look at the statistical model identification. IEEE Transactions on automatic control.

[43] Schwarz G (1978). Estimating the dimension of a model. The Annals of statistics.

[44] Wagenmakers E, Farrell S (2004). AIC model selection using Akaike weights. Psychonomic Bulletin & Review.

[45] Patterson GH, Piston DW (2000). Photobleaching in two-photon excitation microscopy. Biophysical Journal.

[46] Eggeling C, Widengren J, Rigler R, Seidel CAM (1998). Photobleaching of fluorescent dyes under conditions used for single-molecule detection: Evidence of two-step photolysis. Analytical Chemistry.

[47] Widengren J, Rigler R (1996). Mechanisms of photbleaching investigated by fluorescence correlation spectroscopy. Bioimaging.

[48] Xu C, Zipfel W, Shear JB, Williams RM, Webb WW (1996). Multiphoton fluorescence excitation: New spectral windows for biological nonlinear microscopy. Proc. Natl. Acad. Sci. USA.

[49] Hopt A, Neher E (2001). Highly nonlinear photodamage in two-photon fluorescence microscopy. Biophysical Journal.

[50] König K, Becker TW, Fischer P, Riemann I, Halbhuber KJ (1999). Pulse-length dependence of cellular response to intense near-infrared laser pulses in multiphoton microscopes. Optics Letters.

[51] Loomis WF, Lipmann F (1948). Reversible inhibition of the coupling between phosphorylation and oxidation. Journal of biological chemistry.

[52] Blacker TS, Marsh RJ, Duchen MR, Bain AJ (2013). Activated barrier crossing dynamics in the non-radiative decay of NADH and NADPH. Chemical Physics.

[53] König K, So PTC, Mantulin WW, Tromberg BJ, Gratton E (1996). Two-photon excited lifetime imaging of autofluorescence in cells during UVA and NIR photostress. Journal of Microscopy.

[54] Blacker TS (2014). Separating NADH and NADPH fluorescence in live cells and tissues using FLIM. Nature Communications.

[55] Mücksch J, Spielmann T, Sisamakis E, Widengren J (2015). Transient state imaging of live cells using single plane illumination and arbitrary duty cycle excitation pulse trains. Journal of Biophotonics.

[56] Spielmann T, Xu L, Gad AKB, Johansson S, Widengren J (2014). Transient state microscopy probes patterns of altered oxygen consumption in cancer cells. FEBS Journal.

[57] Bresnahan WT, Elving PJ (1981). Spectrophotometric investigation of products formed following the initial one-electron electrochemical reduction of nicotinamide adenine-dinucleotide (NAD. Biochimica et Biophysica Acta.

