# Supplementary material for: Local redox conditions in cells imaged via non-fluorescent transient states of NAD(P)H
Source: Sci Rep. 2019 Oct 21;9:15070. doi: 10.1038/s41598-019-51526-w (PMC6803634; doi:10.1038/s41598-019-51526-w)
Supplement: Supplementary file 1 — Supplementary Information [file 41598_2019_51526_MOESM1_ESM.pdf]

Supplementary Information

Local redox conditions in cells imaged via non-fluorescent transient states of NAD(P)H

Johan Tornmalm[1], Elin Sandberg[1], Mihailo Rabasovic[2], Jerker Widengren[1,*]

[1] Experimental Biomolecular Physics, Department of Applied Physics, Royal Institute of Technology (KTH), Albanova University Center, 106 91 Stockholm, Sweden

[2] Institute of Physics, University of Belgrade, Pregrevica 118, Belgrade 11080, Serbia

1 ELECTRONIC STATE MODEL

The local electronic state probability distribution of fluorophores in a sample can be described by the state vector $\bar{P}(\bar{r},t)$. In the case of the NADH model in Figure 2, the state vector is

$$\bar{P}(\bar{r},t) = \begin{bmatrix} {}^{0}\mathrm{NADH}(\bar{r},t) \\ {}^{1}\mathrm{NADH}(\bar{r},t) \\ {}^{T}\mathrm{NADH}(\bar{r},t) \\ \cdot NADH^{+}(\bar{r},t) \\ NAD^{+}(\bar{r},t) \end{bmatrix} \tag{S1}$$

, with the initial condition that $\bar{P}(\bar{r},0) = [1 \quad 0 \quad 0 \quad 0 \quad 0]^T$ at onset of excitation. All transition rates between states are summarized in the model matrix

$$M(\bar{r}) = \begin{bmatrix} -k_{01}(\bar{r}) & k_{10} & k_T & k_{red} & 0 \\ k_{01}(\bar{r}) & -(k_{10} + k_{isc} + k_{ee}) & 0 & 0 & 0 \\ 0 & k_{isc} & -k_T & 0 & 0 \\ 0 & k_{ee} & 0 & -(k_{red} + k_{deprot}) & 0 \\ 0 & 0 & 0 & k_{deprot} & 0 \end{bmatrix} \tag{S2}$$

, such that the time evolution of the state vector becomes

$$\frac{d\bar{P}(\bar{r},t)}{dt} = M(\bar{r}) \cdot \bar{P}(\bar{r},t) \tag{S3}$$

The one-photon excitation (OPE) rate is calculated as $k_{01}(\bar{r}) = \sigma_{OPE} \cdot \Phi_{exc}(\bar{r})$, where σ_{OPE} is the fluorophore OPE cross section and $\Phi_{exc}(\bar{r})$ is the local excitation photon flux (related to I_{exc} by $\Phi_{exc} = I_{exc}\lambda/(hc)$, where hc/λ is the excitation photon energy). A measurement of the free NADH fluorescence lifetime, $\tau_F = 0.4$ ns, is also used to fix the value for the sum of the first excited singlet state decay rates, $k_{10} + k_{isc} + k_{ee} = 1/\tau_F$.

Note that for rectangular excitation pulses of constant laser intensity, the model matrix $M(\bar{r})$ is time independent during excitation. This allows for efficient calculation of $\bar{P}(\bar{r},t)$ using the matrix exponent

$$\bar{P}(\bar{r},t) = e^{M(\bar{r})t} \cdot \bar{P}(\bar{r},0) \tag{S4}$$

, which can be implemented numerically or worked out analytically by diagonalizing $M(\bar{r})$.

For most fluorophores, including NADH, only the first excited singlet state contributes significantly to the overall luminescence. In the case of a homogeneous sample, the detected luminescence is therefore

$$F(t) = cq_f q_D k_{10} \iiint \left(CEF(\bar{r}) \cdot {}^{1}\mathrm{NADH}(\bar{r},t)\right) dV \tag{S5}$$

, where $CEF(\bar{r})$ is the collection efficiency function of the detection system, q_D the overall detection quantum yield, c is the fluorophore concentration and q_f the fluorescence quantum yield. For a sample with multiple emissive states, the above equation is easily modified to include additional components of the state vector.

2 Average two-photon excitation rate (micro-time averaging)

Two-photon experiments used a Ti:Sapphire excitation laser with a repetition rate of $f_{rep} = 76 \text{ MHz}$ and a pulse width measured to 150 fs FWHM. Since the pulse period ($T = 1/f_{rep} \approx 13 \text{ ns}$) is orders of magnitude shorter than the shortest TRAST pulse duration to be measured (2 µs), the excitation source can be treated as a continuous laser with an average excitation rate of

$$k_{01}(\bar{r},t) = \frac{1}{2T}\sigma_{TPE}\int_{0}^{T}\left(\Phi_{exc}(\bar{r},t,t')\right)^2 dt' \qquad (S6)$$

, where $\Phi_{exc}(\bar{r},t,t')$ describes the local excitation photon flux and σ_{TPE} is the TPE cross section. The integration variable t' refers to micro-time in relation the laser sync signal and is used to describe the excitation dependence within a single laser pulse period. Slower excitation dependencies introduced by TRAST modulation (such as laser scanning, see section 3 in SI) are described by the macro-time, t.

3 Scanned TRAST Gaussian pulse correction (macro-time averaging)

Performing scanned TRAST with a Gaussian excitation laser adds a time dependence to the excitation rate, $k_{01}(\bar{r},t)$, with the irradiance varying continuously as the beam moves past a fixed point in the sample. These variations occur on the same time scale as the intended TRAST pulse duration and must therefore be taken into account through a time-dependent model matrix, $M(\bar{r},t)$. As a result, determining $F(t)$ as outlined in eqs. (S3-S5) becomes considerably more computationally expensive. In this work, we treated scanned TRAST as if the excitation pulses were rectangular in time, thereby allowing the same treatment as for the case of a stationary modulated laser (section 1 in SI).

We thus determined effective rectangular pulse amplitudes and durations that best match the experimental Gaussian pulses (Figure S1C). Simulation of the complete, time-dependent scenario showed that errors introduced by assuming rectangular pulses are minimized for effective pulse amplitudes of 0.71 ± 0.012 (95 % conf.) times the Gaussian peak amplitude and pulse durations of 0.90 ± 0.038 (95 % conf.) times the Gaussian e^{-2} pulse width (Figure S1B).

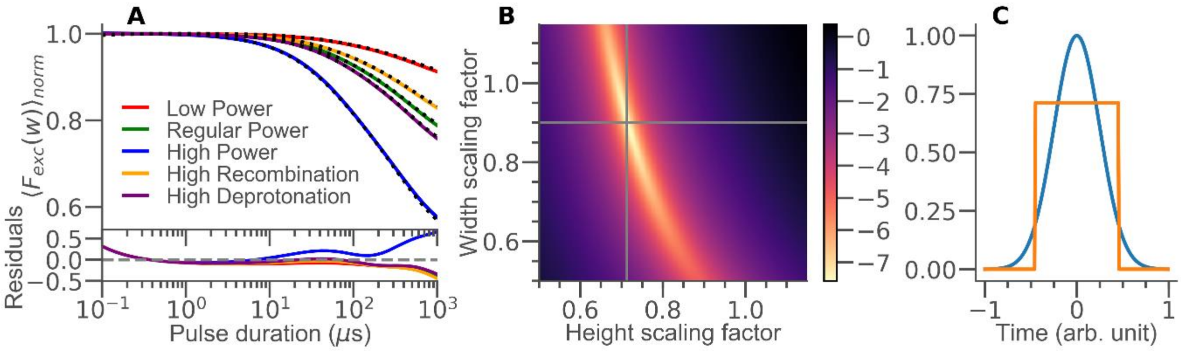

Supplementary Figure S1: *Comparison of rectangular and Gaussian shaped excitation pulses.* ***(A)*** *Simulated TRAST curves from NADH, using different photophysical rate parameters. Coloured curves show simulations based on optimal rectangular pulses. Dashed black lines show corresponding simulations using Gaussian pulse shapes. Absolute residuals shown multiplied by a factor of 100 for clarity.* ***(B)*** *Overall error (RSS, log scale) introduced by the rectangular pulse approximation from all simulated TRAST curves in A, for varying values of the effective pulse height and duration. Optimal parameters indicated by grey cross.* ***(C)*** *Optimal rectangular pulse (amplitude 0.71 and width 0.90) to replace a Gaussian excitation pulse (amplitude 1 and $1/e^2$ width 1).*

The pulse optimization was performed for two very different photophysical models, representing NADH and Rhodamine 110 [1] respectively. Both models returned the same pulse correction factors, irrespective of what photophysical rate parameters were used within those models (Figure S1A, only data for NADH shown). It can be noted, within the given error margins, that the effective pulse amplitude found through simulations corresponds to the fluorescence intensity weighted average amplitude of a Gaussian pulse far from saturation ($k_{01}(\bar{r},t) \ll k_{10}$).

$$k_{01}(\bar{r}) = \frac{\int_{-\infty}^{\infty} \frac{k_{01}(\bar{r},t)}{k_{01}(\bar{r},t) + k_{10}} \cdot k_{01}(\bar{r},t)\, dt}{\int_{-\infty}^{\infty} \frac{k_{01}(\bar{r},t)}{k_{01}(\bar{r},t) + k_{10}} dt} \approx \frac{\int_{-\infty}^{\infty} k_{01}{}^{2}(\bar{r},t)\, dt}{\int_{-\infty}^{\infty} k_{01}(\bar{r},t)\, dt} = \frac{1}{\sqrt{2}} k_{01,peak}(\bar{r}) \qquad (S7)$$

, where $k_{01}(\bar{r},t) = k_{01,peak}(\bar{r}) \cdot e^{-2\left({t}/{\omega_t(\bar{r})}\right)^2}$ is the Gaussian excitation rate. Here $\omega_t(\bar{r}) = \omega_0(\bar{r})/v$ represents the duration of the Gaussian excitation pulse and is directly related to the spatial e^{-2} beam width in the scanning direction, $\omega_0(\bar{r})$, and to the scanner speed, v. For such a rectangular pulse to preserve the total laser irradiance applied, i.e. for the area under the excitation pulse to stay the same, the effective pulse duration is

$$w_{eff}(\bar{r}) = \frac{1}{k_{01}(\bar{r})} \int_{-\infty}^{\infty} k_{01}(\bar{r},t)\, dt = \sqrt{\pi}\omega_t(\bar{r}) \approx 0.89 \cdot \frac{2\omega_0(\bar{r})}{v} \qquad (S8)$$

, again matching the simulated result.

4 Diffusion modelling

Equation (S3) models electronic state transitions but does not take the effects of diffusion into account. A more complete description can be based on the reaction-diffusion equation

$$\frac{d\bar{P}(\bar{r},t)}{dt} = M(\bar{r}) \cdot \bar{P}(\bar{r},t) - D\nabla^2 \bar{P}(\bar{r},t) \qquad (S9)$$

, where D is the diffusion coefficient which in this case was assumed to be constant for all species. Diffusion can speed up dark state recovery by exchanging dark molecules for fresh ones from outside the excitation volume, thereby reducing the observed TRAST amplitude. Since dark states with relaxation times much faster than the diffusion time through the detection volume are not affected in this way [2], it is often sufficient to use a simplified diffusion model based on eq. (S3) with effective diffusion recovery rates inserted in the model matrix $M(\bar{r})$ instead.

$$M(\bar{r}) = \begin{bmatrix} -k_{01}(\bar{r}) & k_{10} & k_T & k_{red} & k_{diff1} & k_{diff2} \\ k_{01}(\bar{r}) & -(k_{10} + k_{isc} + k_{ee}) & 0 & 0 & 0 & 0 \\ 0 & k_{isc} & -k_T & 0 & 0 & 0 \\ 0 & k_{ee} & 0 & -(k_{red} + k_{deprot}) & 0 & 0 \\ 0 & 0 & 0 & k_{deprot} & -(k_{diff1} + k_{diff3}) & 0 \\ 0 & 0 & 0 & 0 & k_{diff3} & -k_{diff2} \end{bmatrix} \qquad (S10)$$

In this work we used the model matrix in eq. (S10), where the faster states are not directly affected by diffusion, but the long-lived NAD^+ is allowed a set of two effective diffusion recovery pathways back to ^{0}NADH. The reason to include two recovery relaxations is that the extended confocal detection volumes are not spherical, but elongated along the optical axis, and thus a slower recovery along this axis might need to be considered. These recovery rates reflect that the depletion zones of ^{0}NADH go beyond the excitation

volumes and depend on the different excitation geometries under OPE and TPE respectively. The diffusion recovery rates were fitted globally for the OPE and TPE measurement data and the values are presented below in Supplementary Table 1.

The second diffusion recovery state, based on k_{diff2} and k_{diff3}, was found to be necessary to fit the OPE data. The corresponding TPE measurements did not require this additional dark state, likely reflecting the more confined excitation volume of TPE. The inclusion of these rates (k_{diff2} and k_{diff3}) also in the TPE global fit resulted in a second diffusion recovery state of near zero lifetime ($1/k_{diff2} \approx 0$), while offering no improvement in fit quality.

Supplementary Table S1: *Fitted diffusion recovery rates for OPE and TPE using the diffusion model in eq. (S10). Uncertainties represent 95 % confidence intervals for the global TRAST fit.*

Rate	OPE	TPE
k_{diff1}	$20 \pm 2\ \mathrm{ms}^{-1}$	$27 \pm 4\ \mathrm{ms}^{-1}$
k_{diff2}	$8 \pm 0.4\ \mathrm{ms}^{-1}$	–
k_{diff3}	$3 \pm 1\ \mathrm{ms}^{-1}$	–

5 SPATIALLY AVERAGED EXCITATION RATE

Typically, a gradient based diffusion model, see eq. (S9), is not required [2, 3]. The calculation of $F(t)$ in eqs. (S1-S5) can then be simplified considerably by removing the spatial dependence altogether. The average sample brightness becomes

$$F(t) = c q_f q_D \cdot {}^1\mathrm{NADH}(t) \tag{S11}$$

, where ${}^1NADH(t)$ is calculated using a space-invariant model matrix, M. The excitation rate $k_{01}(\bar{r})$ is replaced by the average observed excitation rate. For stationary TRAST we use a weighted volume average [4, 5]

$$\bar{k}_{01} = \frac{\iiint k_{01}(\bar{r})\ {}^1\overline{NADH}(\bar{r})\ CEF(\bar{r})\ dV}{\iiint\ {}^1\overline{NADH}(\bar{r})\ CEF(\bar{r})\ dV} \tag{S12}$$

, where ${}^1\overline{NADH}(\bar{r}) = k_{01}(\bar{r})/(k_{01}(\bar{r}) + k_{10})$ is the S_1 population at the onset of excitation, after equilibrium of the singlet states, but before the build-up of dark states. For scanned TRAST the spatial averaging is performed only in the two dimensions perpendicular to the scan direction

$$\bar{k}_{01} = \frac{\iint k_{01}(\bar{r})\ {}^1\overline{NADH}(\bar{r})\ CEF(\bar{r})\ dS}{\iint\ {}^1\overline{NADH}(\bar{r})\ CEF(\bar{r})\ dS} \tag{S13}$$

, since the scan direction now effectively acts as the temporal domain. The averaging along the scan direction was discussed further in SI section 3.

6 DUTY CYCLE CONSIDERATIONS

In the TRAST analysis we assume that all pulses in a pulse train produce an identical fluorescence signal, $F(t)$. In other words, the duty cycle must be low enough to allow a complete return to the same initial distribution, $\bar{P}(\bar{r}, 0)$, between each pulse. In previous works based on TRAST, a fixed duty cycle of $\eta = 0.01$ was proven low enough for the organic fluorophores used [2, 4, 5].

The influence of duty cycle in this study was tested by repeatedly measuring NADH for a few fixed pulse durations while varying the duty cycle. For stationary TRAST (Figure S2A), as the duty cycle approaches $\eta = 1$ (continuous excitation without recovery) all pulse durations obviously produce the same fluorescence signal, while for low enough duty cycles there is no dependence on duty cycle at all. When measuring the OPE TRAST curves in this work (Figure 1), as a compromise between low duty cycle (less distortion) and long illumination time (less noise), we performed our measurements at $\eta_{OPE} = 0.01$.

For scanned TRAST measurements there is no connection between measurement time and duty cycle. Instead, we are free to select arbitrarily low duty cycles by increasing the length of the path to be scanned. In this case we used a duty cycle of $\eta_{TPE} = 0.0046$, below the region where we expect distortions in the TRAST curves. As shown in Figure S2B, there is no change in the data if the duty cycle is lowered further.

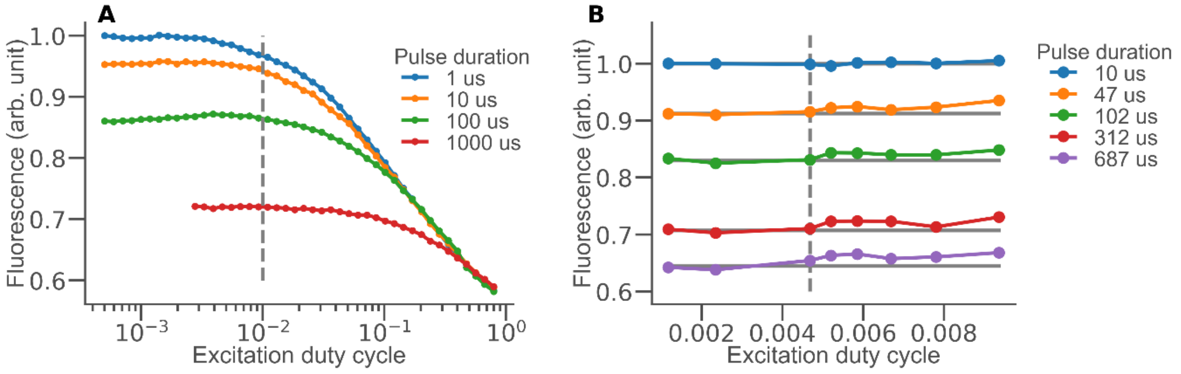

Supplementary Figure S2: *Normalized fluorescence from TRAST measurements with different pulse durations and duty cycles. The dashed grey lines mark the duty cycle used for experiments described in the main text.* ***(A)*** *$1\ \mu M$ NADH in 50 mM TRIS buffer, pH 7.4, measured using OPE stationary TRAST.* ***(B)*** *$10\ \mu M$ NADH in 50 mM TRIS buffer, pH 7.4, measured using TPE scanned TRAST.*

7 SCANNED TRAST SAMPLE DATA

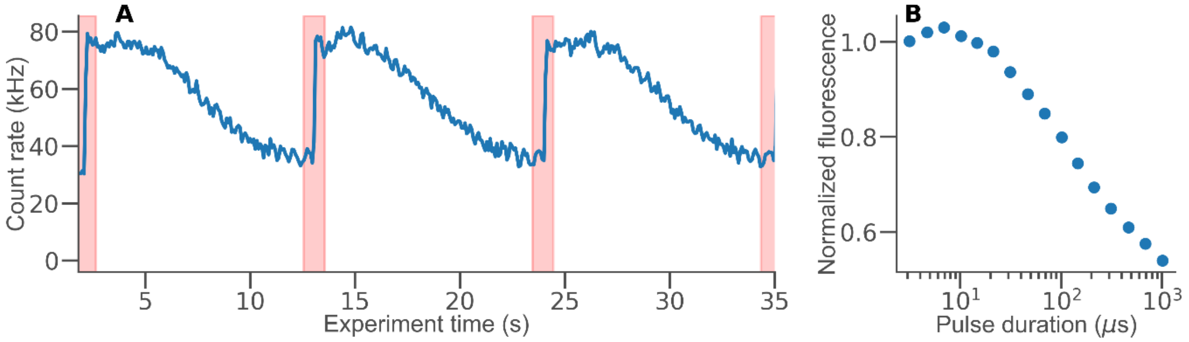

Supplementary Figure S3: (A) Typical time trace acquired during TPE scanned TRAST measurements of NADH in Tris buffer (50 mM, pH 7.4) solution. The laser is scanning a circle of 28.3 μm diameter and the speed is varied such that the effective dwell time ranges from 3 μs to 1 ms (see effective pulse duration in SI section 3). As the scan speed is lowered, the steady state count rate goes down as the population of photo-oxidized NADH increases. The figure shows the experiment repeated three times in quick succession, with no visible photobleaching of the sample. The red areas mark where the scanner speed was reset from slowest to fastest, making the speed unstable. These areas are excluded from the TRAST analysis. **(B)** Corresponding TRAST curve showing normalized fluorescence intensity as a function of effective laser dwell time, calculated by combining the data from the raw trace shown in A.

8 Model evaluation for OPE (AIC/BIC)

A set of similar photophysical models were compared by use of both the Akaike information criterion (AIC) and the Bayesian information criterion (BIC) [6]. For least squares fitting of multiple models to the same data, AIC and BIC scores can be calculated by

$$AIC = n\ln(RSS) + 2k$$
$$BIC = n\ln(RSS) + k\ln(n)$$

, where RSS is the residual sum of squares from the fit, n the number of data points (constant) and k is the number of fitted parameters in the model. The model with the lowest AIC/BIC score is preferred. The two criteria differ only in how much they penalise models with a larger number of fit parameters, k. In our case, with a relatively large n, the BIC is much more restrictive towards additional model parameters.

If the lowest AIC score is AIC_{min} and model i deviates from this score by $\Delta_i(AIC) = AIC_i - AIC_{min}$, the relative likelihoods of each tested model can be expressed by the Akaike weights

$$w_i(AIC) = \frac{e^{-\frac{1}{2}\Delta_i(AIC)}}{\sum_{k=1}^{K} e^{-\frac{1}{2}\Delta_k(AIC)}}$$

, where K is the total number of models tested. Weights for the BIC scores can be calculated in the same way. Figure S4 shows the calculated weights for both AIC and BIC for 9 different variations of the model in Figure 2, with this base model receiving by far the highest likelihood according to both criteria. The BIC strongly prefers models with fewer parameters and assigns also a small probability to a model without any triplet state at all (M1). The AIC is more permissive towards additional rates and suggests small probabilities also for electron ejection from the triplet state (M2) and photon driven electron ejection from ^1NADH (M4).

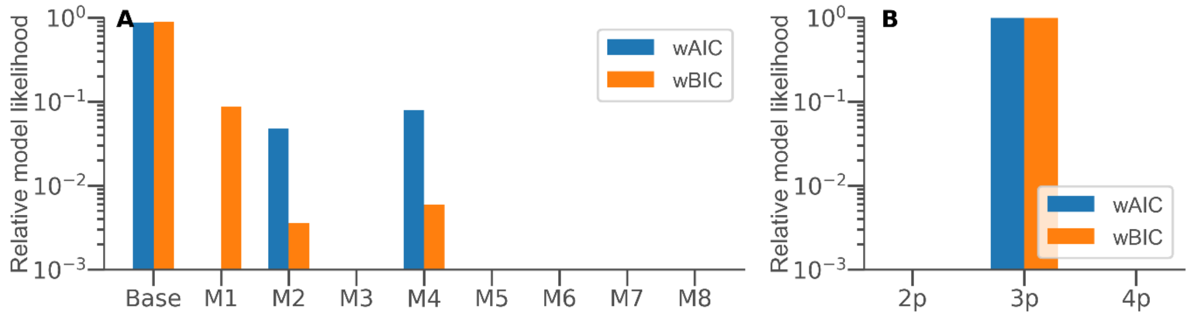

Supplementary Figure S4: ***(A)*** *Relative likelihoods ($w_i(AIC)$ and $w_i(BIC)$) for the base model defined by eq. (S10) and illustrated in Figure 2, as well as 8 variations on this model. Weights were calculated based on global fits of all OPE TRAST curves. Model* ***M1*** *includes no triplet state. Model* ***M2*** *includes an additional rate for electron ejection from the triplet state directly to* $\cdot NADH^+$. ***M3*** *has a photon driven electron ejection from ^1NADH to $\cdot NADH^+$, such that the whole electron ejection process from the ground state shows a two-photon dependence.* ***M4*** *combines both a spontaneous electron ejection from ^1NADH (one-photon dependence) with a photon driven electron ejection (two-photon dependence).* ***M5*** *lets oxygen influence k_{deprot} instead of k_{red}.* ***M6*** *lets H_2O_2 influence the diffusion recovery rate k_{diff1} instead of k_{deprot}.* ***M7*** *includes only one effective diffusion recovery component. The slowest state in (S10) is removed.* ***M8*** *fixes k_{diff2} to zero to emulate permanent bleaching without recovery.* ***(B)*** *Relative likelihoods ($w_i(AIC)$ and $w_i(BIC)$) for TPE NADH models (Figure 2) with either a two-, three-, or four-photon dependence on the overall electron-ejection rate. The preferred three-photon model consists of TPE from ^{0}NADH to ^{1}NADH and then a subsequent OPE from ^{1}NADH to ^{n}NADH where the electron-ejection yield is higher.*

9 CLASSIFICATION PROBABILITIES FOR SINGLE CELLS

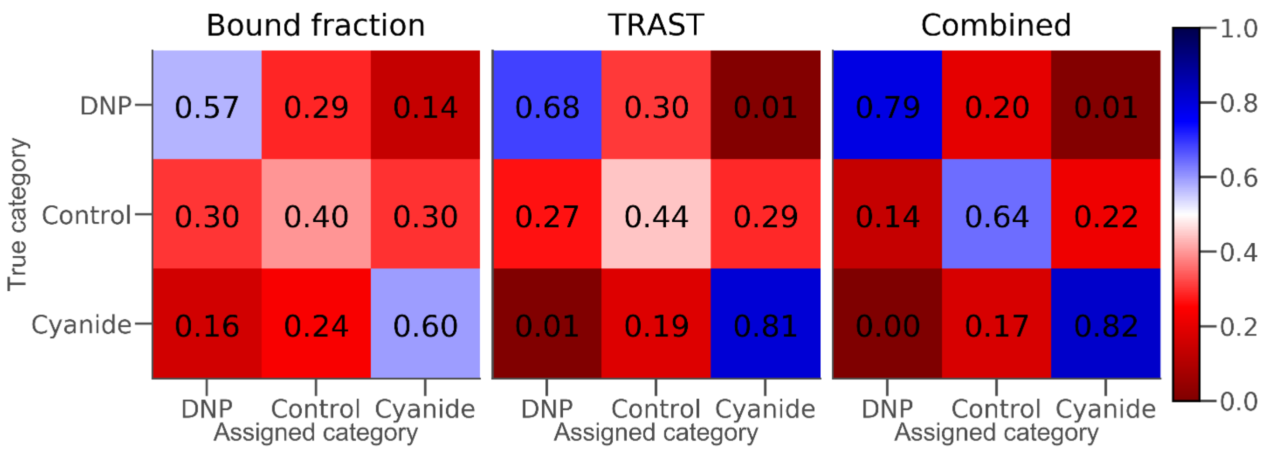

Supplementary Figure S5: *Average classification probabilities for single cells, obtained from the machine learning algorithm as described in Methods and Materials. Training was performed using either the average bound fraction of NAD(P)H,* $\langle A_{bound} \rangle$*, (left matrix), average dark state amplitude,* $\langle A_{TRAST} \rangle$*, (middle matrix), or a combination of both (right matrix). Values represent the average performance over* 10^3 *training and evaluation runs, each using a new random subset of cells for training (80 % of cells) and evaluation (remaining 20 %).*

References

1. Blom, H., Chmyrov, A., Hassler, K., Davis, L.M. & Widengren, J. Triplet-State Investigations of Fluorescent Dyes at Dielectric Interfaces Using Total Internal Reflection Fluorescence Correlation Spectroscopy. *Journal of Physical Chemistry A* **113**, 5554-5566 (2009).
2. Spielmann, T., Blom, H., Geissbuehler, M., Lasser, T. & Widengren, J. Transient State Monitoring by Total Internal Reflection Fluorescence Microscopy. *Journal of Physical Chemistry B* **114**, 4035-4046 (2010).
3. Mucksch, J., Spielmann, T., Sisamakis, E. & Widengren, J. Transient state imaging of live cells using single plane illumination and arbitrary duty cycle excitation pulse trains. *Journal of Biophotonics* **8**, 392-400 (2015).
4. Chmyrov, V., Spielmann, T., Hevekerl, H. & Widengren, J. Trans-Cis Isomerization of Lipophilic Dyes Probing Membrane Microviscosity in Biological Membranes and in Live Cells. *Analytical Chemistry* **87**, 5690-5697 (2015).
5. Spielmann, T., Xu, L., Gad, A.K.B., Johansson, S. & Widengren, J. Transient state microscopy probes patterns of altered oxygen consumption in cancer cells. *Febs Journal* **281**, 1317-1332 (2014).
6. Wagenmakers, E. & Farrell, S. AIC model selection using Akaike weights. *Psychonomic Bulletin & Review* **11**, 192-196 (2004).
